# Endogenous living adjuvants for cancer vaccination: Concepts, mechanisms, and design principles

**DOI:** 10.1016/j.mtbio.2026.103020

**Published:** 2026-03-10

**Authors:** Jiaxin Yu, Chaojie Zhu, Hongjun Li, Feng Xu

**Affiliations:** a Shaoxing People's Hospital (Shaoxing Hospital, Zhejiang University School of Medicine), Shaoxing, Zhejiang, 312000, China; b School of Medicine, Shaoxing University, Shaoxing, Zhejiang, 312000, China; c State Key Laboratory of Advanced Drug Delivery and Release Systems, Liangzhu Laboratory, School of Pharmacy, Zhejiang University, Hangzhou, China; d Department of Hepatobiliary and Pancreatic Surgery, The Second Affiliated Hospital, School of Medicine, Zhejiang University, Hangzhou, 310009, China; e The First Affiliated Hospital of Shaoxing University, Shaoxing, Zhejiang, 312000, China

**Keywords:** Cancer vaccine, Adjuvant, Immune engineering, Drug delivery, Biomaterials, Living therapeutics

## Abstract

Cancer vaccination requires effective integration of antigen delivery and immune activation across secondary lymphoid organs and tumor tissues, yet many conventional adjuvants rely on static molecular or material-based cues that are poorly aligned with dynamic antitumor immune processes. To address this gap, the concept of living adjuvants has emerged to describe biologically active systems that enhance vaccination by actively participating in immune regulation rather than serving as passive stimulatory components. While the broader concept may include microorganisms, we focus exclusively on endogenous living adjuvants, defined as self-derived cellular systems, including dendritic cells, red blood cells, B cells, mesenchymal stromal cells, and tumor cells, which share key features such as physiological trafficking, sustained cellular interactions, and amenability to chemical or genetic engineering, enabling integrated immune modulation within a single platform. We define the core concepts and mechanisms underlying endogenous living adjuvants, summarize representative strategies, and discuss design principles that govern their effectiveness, controllability, safety, and translational potential. This review provides a unified framework to guide the rational engineering of endogenous living adjuvants, ultimately informing the development of next-generation cancer immunotherapies.

## Graphical abstract

**Figure:**
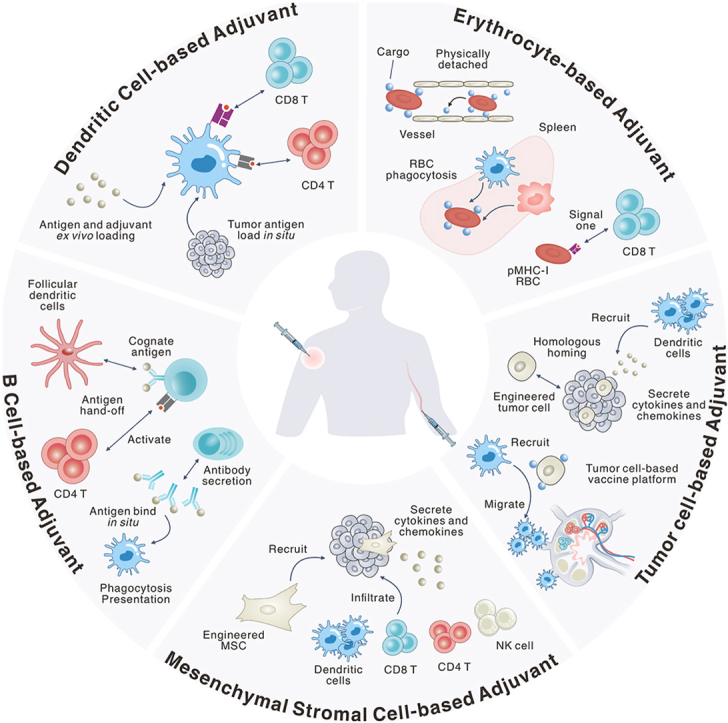

## 1 Introduction

Therapeutic cancer vaccination aims to induce durable and systemic antitumor immunity by directing host immune responses against tumor cells [1]. Unlike prophylactic vaccines for infectious diseases, cancer vaccines must overcome tumor-related immune tolerance, tumor heterogeneity, and a continuously evolving tumor microenvironment [2]. Although advances in antigen discovery and immunological understanding have enabled the induction of measurable vaccine-specific immune responses, clinical benefit remains inconsistent [3,4]. In many settings, antigen-specific T cell responses can be detected after vaccination, yet durable tumor control is achieved only in a subset of patients [5,6]. These observations suggest that the central challenge of cancer vaccination lies not in the ability to initiate immune responses, but in how vaccine systems are designed to produce immune responses of sufficient quality and durability [7].

Initial efforts to improve cancer vaccine efficacy focused on antigen selection, reflecting the central role of antigen specificity in immune recognition [6,8]. Tumor-associated antigens are often weakly immunogenic, shaped by immune tolerance, and susceptible to immune escape [9,10]. Personalized neoantigen vaccines have partially addressed these limitations by increasing antigen specificity [11,12]. However, even with optimized antigens, vaccine-induced immune responses frequently remain transient or functionally suboptimal [2,13]. This has shifted attention from antigen selection to delivery design, specifically how antigens reach secondary lymphoid organs and are subjected to dendritic cell activation and cross-presentation [14,15].

Adjuvant is a critical component of cancer vaccine design. In practice, adjuvant function extends beyond immune stimulation and can be decomposed into two coupled tasks: (1) delivering antigen and immunomodulatory cues to the lymphoid organs and to specific immune cell types (*e.g.*, dendritic cells and B cells), and (2) programming antigen-presenting cells to support adaptive immune response initiation [[16], [17], [18], [19]]. Effective antitumor immunity requires coordinated antigen delivery and immune activation within secondary lymphoid organs, followed by the generation of effector CD8^+^ T cells that can traffic to tumor tissues and sustain cytotoxic function [20,21]. Conventional adjuvants are compositionally defined and controllable, and they are highly effective in many prophylactic vaccines (*e.g.*, alum-, MF59-, or CpG-adjuvanted licensed vaccines) for infectious diseases [[22], [23], [24]], yet they often do not reliably drive the magnitude and cross-priming efficiency needed for tumor-controlling cellular immunity in therapeutic settings [25,26].

**Fig. 1:**
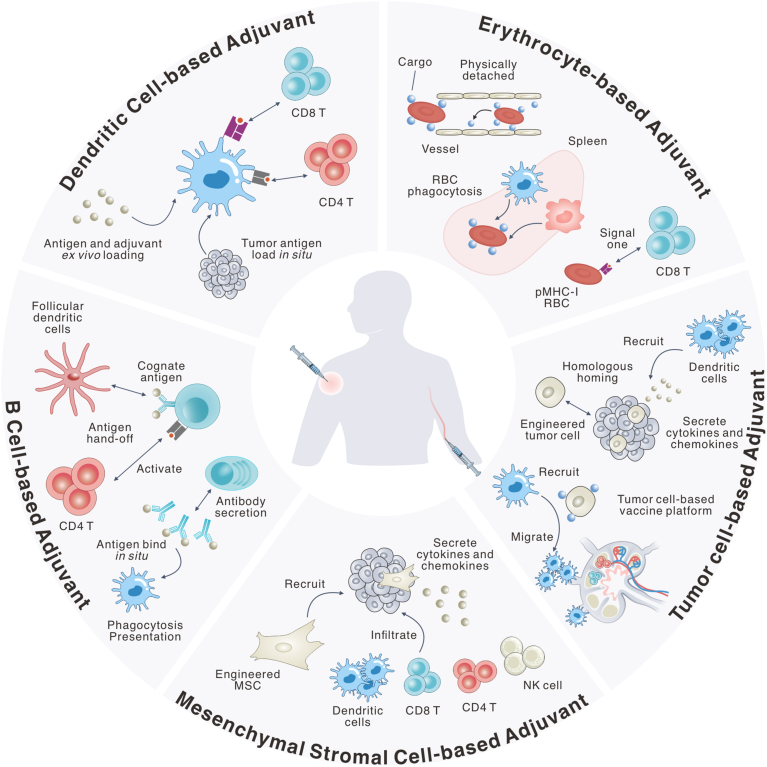
**Schematic illustration of endogenous living adjuvant systems for cancer vaccination.** Dendritic cell platforms are either loaded *ex vivo* with tumor antigens together with innate agonists or costimulatory cues or acquire tumor antigens *in situ* to promote priming of tumor-specific T cells. Erythrocyte-based platforms can be engineered as circulating carriers that deliver immunomodulatory payloads to defined organs (*e.g.*, *via* physiological filtration or vascular-bed targeting) to support local immune activation. RBC can also be functionalized with peptide-MHC class I (pMHC-I) complexes to provide antigen-specific “signal 1” to CD8^+^ T cells. B-cell platforms can present antigen on pMHC-II to activate cognate CD4^+^ T cells, and can handle antigen into follicular niches, including transfer to follicular dendritic cells to support humoral responses. In addition, antibodies produced by engineered B cells can opsonize tumor antigens to form immune complexes, facilitating Fc receptor (FcR)-mediated uptake by DCs and enhancing antigen processing and presentation. Mesenchymal stromal cell platforms can be engineered to secrete cytokines and chemokines or display costimulatory ligands. Upon accumulation in inflamed tissues and tumors, they can bias local immune cell recruitment and activation, including DCs, T cells, and NK cells. Tumor cell-based platforms provide antigen sources and can be further engineered to introduce innate activation signals (*e.g.*, type I interferon-inducing cues) or chemokines within the tumor microenvironment. In some designs, tumor cells are functionalized with immunostimulatory cargos to couple antigen release with local immune activation and elicit antitumor immunity.

These considerations have motivated interest in living adjuvants, broadly defined as biologically active cellular-based systems that participate directly in immune regulation [27]. In principle, living adjuvants may include a wide range of platforms, from host-derived cells to microorganisms such as bacteria, fungi, and even viruses [28,29]. In this review, we focus specifically on endogenous living adjuvants, host-derived cellular systems, including dendritic cells, red blood cells, B cells, mesenchymal stromal cells, and tumor cell platforms (Fig. 1). Mechanistically, endogenous living adjuvants expand cancer vaccine design in two dimensions. Endogenous living adjuvants can serve as cellular antigen carriers that exploit endogenous trafficking routes, including lymphatic drainage and CCR7-dependent lymph node homing, thereby increasing antigen access to secondary lymphoid organs and facilitating cross-presentation. Beyond transport, these systems leverage inherent intercellular communication, such as receptor-ligand interactions and regulated paracrine secretion of cytokines and chemokines, rather than relying solely on exogenous pattern-recognition receptor (PRR) agonists.

To establish a rigorous framework, we define endogenous living adjuvants as viable, host-derived cellular systems that actively participate in at least one core adjuvant function, (1) antigen delivery to lymphoid organs, (2) innate immune activation, or (3) programming of adaptive responses, through their intrinsic biological processes (physiological trafficking, regulated intercellular signaling, or persistence). Within this framework, different cellular platforms occupy distinct functional niches along a spectrum from anatomical delivery to active immune instruction. For instance, red blood cells primarily sit at the delivery end of the spectrum, serving as stealthy, long-circulating vehicles that exploit physiological clearance pathways to shuttle antigens to splenic phagocytes. Conversely, dendritic cells and B cells operate at the instructional end, directly orchestrating T cell priming and follicular responses *via* active antigen presentation and costimulation. Meanwhile, mesenchymal stromal cells and engineered tumor cells function as microenvironmental modulators, positioning themselves in the middle of the spectrum to provide localized cytokine conditioning and spatial coordination of immune cells.

In addition, the minimal requirements for a platform to qualify as an endogenous living adjuvant are: (1) retention of cellular viability and biological activity during the critical immune engagement environment (2) active contribution *via* endogenous mechanisms (*e.g.*, tissue homing, immunomodulatory protein secretion, or cell-cell contact); that cannot be fully replicated by non-viable chassis; and (3) amenability to chemical or genetic engineering for improved efficacy, controllability, and safety. Borderline examples are explicitly distinguished. Inactivated or non-viable tumor cell preparations (*e.g.*, irradiated or cryo-shocked tumor cells discussed in Section 3.5) provide valuable antigen sources and trafficking cues but lack cellular viability, thus falling outside this definition and instead serving as crucial supplements to endogenous living adjuvants. By integrating mechanistic insights with emerging engineering strategies, we aim to clarify how endogenous living adjuvants expand the design space of cancer vaccines beyond conventional formulations and inform the rational development of next-generation cancer vaccination strategies.

## 2 Immunological requirements of cancer vaccination

### 2.1 Defined-antigen cancer vaccination

**Fig. 2:**
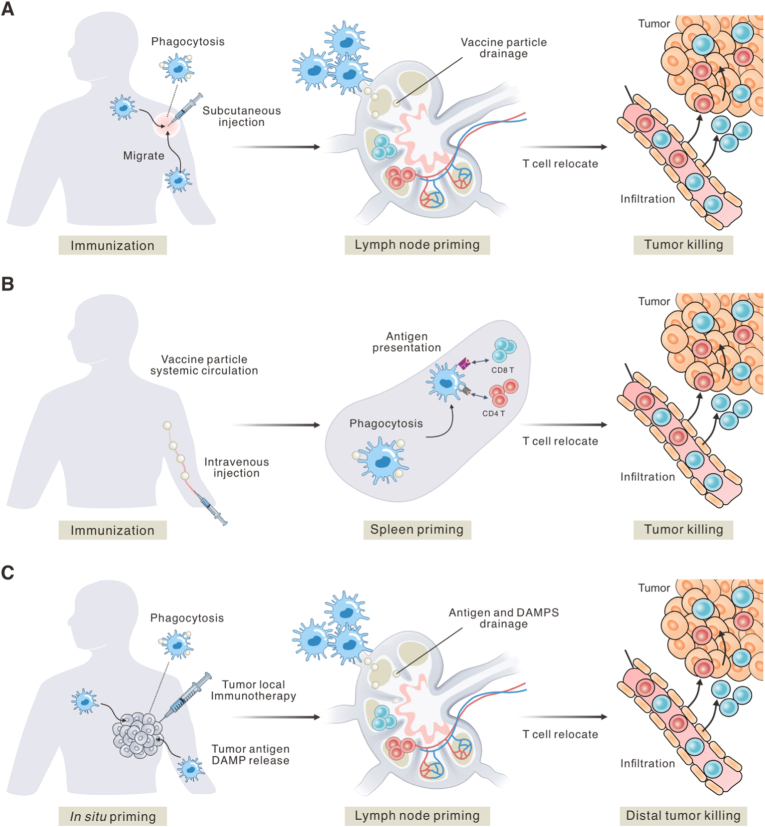
**Schematic illustration of therapeutic mechanisms of cancer vaccination. (A)** Subcutaneous injection of vaccine formulation to elicit the antitumor immune response. **(B)** Intravenous injection of vaccine formulation for initiating spleen-centered antitumor immune response. **(C)***In situ* vaccination efficacy to exert systemic immune response against both local and distal tumors.

Defined-antigen cancer vaccines commonly prime T cells through secondary lymphoid organs [2], while *in situ* vaccination can enter the same lymphoid circuits after tumor antigen release and may additionally engage tertiary lymphoid structures in some tumors [30,31]. For defined-antigen vaccination, delivery design determines whether antigen reaches draining lymph nodes in a lymphatic-dominant manner, is carried there by migrating antigen-loaded cells, or enters the bloodstream to engage splenic circuits [32,33]. After subcutaneous or intramuscular injection, small soluble antigens and appropriately sized particulate carriers can drain through afferent lymphatics and accumulate in the draining lymph node (Fig. 2A) [34]. Vaccine particles are first encountered in the subcapsular sinus by CD169^+^ macrophages and resident antigen-presenting cells and are then relayed to intranodal dendritic cell networks [35,36]. In parallel, the injection site recruits dendritic cells and monocytes that internalize antigen locally. These cells upregulate CCR7 and home to the draining node along CCL19 and CCL21 gradients [37]. Carrier physicochemical properties bias these routes. Faster lymphatic access favors small, neutrally or mildly anionic species and nanoparticles that avoid rapid local sequestration, whereas highly opsonized or larger particles are more likely to be retained in tissue and rely on cell-mediated transport [15,38].

Delivery also determines which APC subsets are engaged. Surface chemistry and ligands can shift uptake toward dendritic cells rather than non-professional phagocytes [39,40], and intracellular trafficking features that favor cytosolic access increase the probability of cross-presentation [40]. When vaccine particles enter the bloodstream, splenic priming becomes prominent (Fig. 2B). In the spleen, resident cDC2 are enriched in marginal zone bridging channels and can upregulate CCR7 to relocate toward the white-pulp T-cell zone after immunization, whereas cDC1 preferentially localize within the periarteriolar lymphoid sheath (PALS) and are specialized for cross-presentation once blood-borne antigen is transported from the marginal zone into the white pulp [[41], [42], [43]]. Intravenously administered RNA-lipoplexes provide preliminary evidence, as their formulation drives spleen-centered uptake and antigen expression in dendritic cell populations together with a type-I IFN program, aligning blood-borne delivery with splenic cross-priming [44].

Apart from delivery, the immunomodulatory profile of the adjuvant can determine the type and magnitude of the elicited adaptive immune responses. Innate sensing through PRRs activates MyD88-and TRIF-dependent pathways and induces NF-κB and IRF transcriptional programs, driving dendritic cell maturation with increased expression of MHC, CD80, CD86, and CD40 [45]. Cross-presenting cDC1 (BATF3-IRF8-dependent, XCR1^+^) route exogenous antigens into the MHC-I pathway through cytosolic processing and TAP-dependent peptide loading, generating stable peptide-MHC-I display for CD8^+^ T-cell recognition [46]. T-cell priming follows a compact signal logic: TCR recognition of peptide-MHC-I provides signal 1, CD28 engagement by CD80 and CD86 provides signal 2, and cytokine modulation dominated by type-I IFN and IL-12 provides signal 3, with CD40^−^CD40L interactions reinforcing sustained cross-priming [47,48].

In the phase I study of the individualized mRNA neoantigen vaccine autogene cevumeran in resected pancreatic ductal adenocarcinoma, vaccine-induced high-magnitude neoantigen-specific T-cell responses were detected in 8 of 16 patients and were associated with delayed recurrence relative to non-responders [49]. This result is consistent with a recurrent limitation in therapeutic vaccination: antigen specification is necessary, but delivery into the appropriate lymphoid priming niches and consistent dendritic cell cross-priming at tumor-controlling magnitude remain difficult to achieve across patients [50].

### 2.2 *In situ* cancer vaccination

*In situ* vaccination differs fundamentally from conventional vaccination in that antigens are not predefined or pre-formulated, but are generated endogenously within tumor tissues. Local interventions such as intratumoral immunostimulants, oncolytic viruses, radiotherapy, or certain chemotherapeutic and ablative approaches can induce tumor cell death and stress responses, leading to the release of a diverse repertoire of tumor-associated antigens [[51], [52], [53], [54]]. These antigens are then processed within the tumor microenvironment and can initiate immune responses that extend immune cytotoxicity beyond the treated lesion [55]. From an immunological perspective, *in situ* vaccination places the tumor tissue itself at the center of immune education (Fig. 2C). Antigen-presenting cells operating in this case must integrate antigens with tissue-derived cues, including inflammatory signals, damage-associated molecular patterns (DAMPs), and immunosuppressive factors [56]. This local immune conditioning influences antigen uptake, processing, and the functional state of antigen-presenting cells, which in turn affects their capacity to prime T cells. Unlike lymphoid priming driven by conventional vaccine delivery routes, immune activation *in situ* is tightly coupled to the spatiotemporal immune state of the tumor [57]. Talimogene Laherparepvec (T-VEC), approved for melanoma, exemplifies that viral replication and tumor cell lysis can promote antigen release while simultaneously providing innate immune stimulation [58]. Although clinical responses are often restricted to a subset of patients, T-VEC demonstrates that local immune perturbation can generate systemic antitumor immune effects, supporting the conceptual rationale of *in situ* vaccination.

The central hurdle for *in situ* vaccination, however, stems from the spatiotemporal heterogeneity of the tumor microenvironment. Antigen release is temporally variable and often spatially restricted, occurring preferentially in regions of necrosis, hypoxia, or immune infiltration [59,60]. Antigen-presenting cells recruited to these areas may encounter conflicting signals that shape their phenotype and migratory behavior [61]. Effective *in situ* vaccination, therefore, requires not only antigen availability, but also the permissive environment that support antigen presenting cells’ activation and their subsequent trafficking to lymphoid tissues. Crucially, *in situ* vaccination does not replace lymphoid immune priming, but rather feeds into it [62]. Tumor-educated antigen-presenting cells must ultimately interact with naive or memory T cells within lymph nodes or the spleen to generate durable systemic immunity [63]. In this regard, *in situ* vaccination represents a multi-step immune process that spans tissue and lymphoid compartments. Its success depends on the coordination of local immune education with downstream adaptive immune programming [64]. Together with systemic vaccination, *in situ* vaccination is a complementary immunological route to antitumor immunity.

## 3 Endogenous living adjuvants for cancer vaccination

Adjuvants are classically defined as components of vaccine formulations that enhance immune responses to associated antigens [17]. In practical terms, adjuvant function can be broadly decomposed into two interrelated roles [18]. One is antigen delivery, including antigen protection, transport, and access to antigen-presenting cells within secondary lymphoid organs. The other is immune activation, whereby innate immune signals promote antigen uptake, antigen presentation, and the initiation of adaptive immune responses [65,66]. Most conventional adjuvants achieve these functions through molecular or biomaterial-based designs, such as depot formation, particulate delivery, or engagement of pattern recognition receptors [17].

Endogenous living adjuvants can fulfill these same functions through distinct biological mechanisms. Many cell-based platforms, particularly antigen-presenting cells, are intrinsically capable of carrying antigenic cargo, migrating to lymph nodes or spleen, and directly engaging T cells through organized cell-cell interactions [63]. In these cases, antigen delivery is achieved not by passive transport, but by active cellular trafficking, while immune activation is associated with physiological pathways for antigen presentation. In addition, the biological activity of living systems also makes them amenable to genetic and chemical engineering, allowing their delivery behavior, immunostimulatory properties, and safety profiles to be systematically tuned [67]. Through such interventions, endogenous living systems can be transformed into adaptable platforms that integrate antigen delivery and immune activation within a single entity.

In this section, we examine these endogenous living adjuvants across different cell classes: dendritic cells, erythrocytes, B cells, mesenchymal stromal cells, and tumor cells. For each class, we first discuss the intrinsic biological features that enable adjuvant-like function, followed by representative examples illustrating how genetic or chemical engineering strategies have been applied to enhance cancer vaccination.

### 3.1 Dendritic cell

Dendritic cells are professional antigen-presenting cells specialized for capturing antigens, converting them into peptide-major histocompatibility complex (p-MHC) ligands, and instructing T cell fate through co-stimulation and cytokines [68]. Antigen acquisition in immature DCs is high and relies heavily on constitutive macropinocytosis for soluble antigens, as well as receptor-mediated endocytosis *via* lectin-type receptors, such as the mannose receptor, and phagocytosis for particulate cargo [68,69]. The uptake route matters because it determines intracellular routing and the potential for cross-presentation. Macropinocytosis is typically downregulated during maturation, whereas mature DCs can continue to capture antigens preferentially through endocytic receptors and phagocytosis rather than bulk fluid uptake [70]. Immature DCs are therefore optimized for antigen capture but express low levels of costimulatory molecules, while mature DCs upregulate co-stimulation and antigen presentation capacity [71]. Clinically, the feasibility of autologous APC-based vaccination is exemplified by sipuleucel T, manufactured by leukapheresis followed by *ex vivo* culture of patient PBMCs with PA2024, a fusion protein of prostatic acid phosphatase and GM-CSF [[72], [73], [74]]. Its modest but reproducible survival benefit highlights both the promise and constraints of cell vaccines, including limited functional potency in advanced disease, variability in product and host immunity, and suppression of DC function by tumor-associated factors [5,75]. These features motivate viewing DCs as endogenous living adjuvants, since they can be loaded with antigen and tuned *ex vivo* to enable programmable immune modulation, rather than providing only a passive depot of immunostimulatory cues [[76], [77], [78]].

Building on this clinical foundation, a series of engineering strategies has emerged that explicitly reposition dendritic cells as endogenous living adjuvants. A first class of approaches addresses one of the most persistent limitations of DC vaccination, the inefficient and variable delivery of reinfused cells to secondary lymphoid tissues [79]. Magnetic nanoparticle-based strategies exemplify this direction by enabling both physical guidance and quantitative tracking of DC migration. In seminal studies, dendritic cells labeled with fluorescent magnetic nanoparticles were shown to accumulate preferentially in tumor-draining lymph nodes under external magnetic fields by 5-fold compared with the magnetic-free condition [80]. In EG7-OVA lymphoma models, magnetically-enriched DC vaccines achieved tumor inhibition rates of 96%, compared with 79% in the magnetic-free condition.

**Fig. 3:**
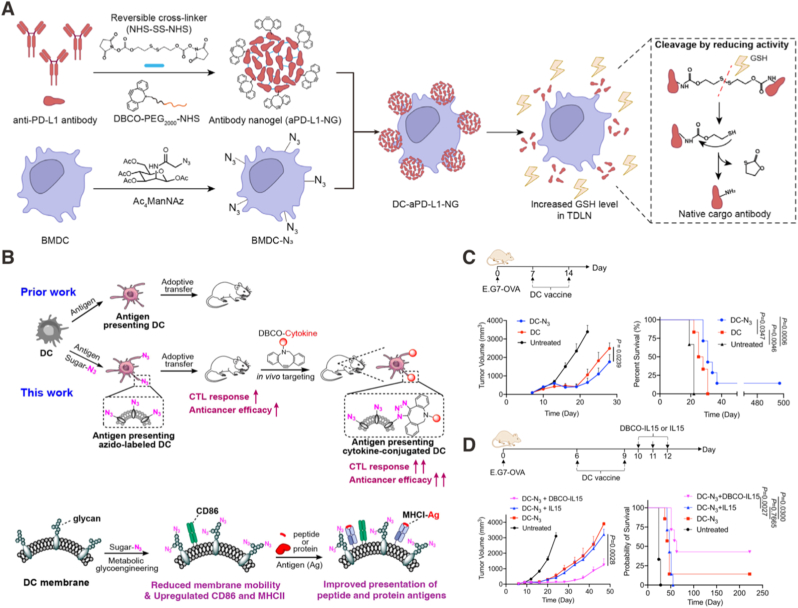
**Engineering strategies for dendritic cell-based living adjuvants in cancer vaccination. (A)** DC surface decoration with an anti-PD-L1 antibody nanogel that undergoes tumor-draining lymph node-responsive release to locally deliver immune checkpoint blockade [83]. Copyright 2025, *© 2025 Wiley‐VCH GmbH*. **(B)** Metabolic glycan labeling of DCs to install azide groups for *in situ*, biorthogonal conjugation of exogenous immunomodulators (*e.g.*, DBCO-IL-15). **(C)** Therapeutic efficacy of DC vaccines with *versus* without glycan labeling. **(D)** Therapeutic efficacy of DC-N3 alone or combined with DBCO-IL-15 or soluble IL-15, compared with an untreated control [84]. Copyright 2023, Springer Nature, *The Author(s).*

Transitioning from systemic delivery to local interactions, subsequent strategies focus on enhancing DC function at the level of immune synapse formation and local signal integration [81]. Rather than administering adjuvants systemically, these approaches physically couple immunomodulatory agents to the DC surface, thereby localizing immune activation to the DC-T cell interface. A representative example is the lymph node-targeted cell-nanoadjuvant conjugate in which R848 (TLR 7/8 agonist)-loaded liposomes and anti-PD1 antibodies were chemically conjugated to DC membranes *via* click reaction before reinfusion [82]. Mechanistically, the sustained release of R848 maintained the immunostimulatory phenotype of the injected DCs and facilitated their accumulation in the lymph node. The surface-linked anti-PD1 antibodies increased DC-T cell adhesion, together amplifying IFN-γ and IL-12 feedback loops. In murine 4T1 metastasis models, this surface-engineered DC platform yielded 40% survival at day 45, whereas no mice survived to day 45 in the other treatment groups. A related concept is illustrated by dendritic cell-nanogel conjugates designed for tumor-draining lymph node (TDLN)-specific PD-L1 blockade [83]. By anchoring immunomodulatory nanogels (NHS-SS-NHS cross-linked anti-PDL1 antibodies functionalized with DBCO-PEG2000-NHS) onto DCs, together with loading tumor antigens, this strategy achieved responsive release of the immune checkpoint inhibitors under a reductive TDLN microenvironment (Fig. 3A). In 4T1 cancer models, this platform achieved 83.3% survival over 60 days with tumor-free, in comparison to all mice dead before day 50 in the control dendritic cell tumor vaccine group.

Concurrently, an increasingly influential direction involves programming DC function from within by delivering nucleic acids that encode both antigens and immunomodulatory agents [85,86]. Compared with peptide pulsing, which provides a finite and progressively declining pool of surface peptide-MHC complexes, mRNA engineering may enable sustained endogenous antigen expression, continuously supplying protein substrates for proteasome-TAP-dependent MHC-I loading and extending the effective window for cross-priming [[87], [88], [89]]. Because the translated product can be a full-length antigen or a multi-epitope construct, this approach reduces HLA pre-restriction and can support concurrent MHC-I and MHC-II presentation, thereby strengthening coordination between CD8^+^ effector differentiation and CD4^+^ T cells’ help [90,91]. Lipid nanoparticle-mediated mRNA delivery provides a feasible route to implement this strategy, while allowing formulation-level control over intracellular trafficking and innate sensing [[92], [93], [94], [95]]. Beyond antigen expression, nucleic-acid programming enables direct modulation of DC regulatory pathways to improve the durability and functional quality of T-cell priming. More advanced designs have integrated dual mRNA and siRNA delivery into DCs, integrating sustained antigen presentation with reinforcement of costimulatory and cytokine production while silencing inhibitory pathways such as PD-L1 and PD-L2 [96,97]. Importantly, because these genetic programs are confined to the adoptively transferred DCs, immune activation remains spatially and temporally constrained, linking potency to a defined cellular compartment rather than diffuse systemic exposure, reinforcing the concept of DCs as programmable living adjuvants rather than indiscriminate immune stimulants.

Finally, recent work has begun to move beyond preloaded antigen paradigms toward dynamic systems that allow DCs to acquire antigen *in vivo* and participate in *in situ* immune education. An illustrative example is the integration of antigen-capturing nanoparticles with migratory type 1 conventional dendritic cell therapy [98]. In this approach, nanoparticles designed to bind tumor-derived antigens within the tumor microenvironment were combined with adoptively transferred cDC1 cells, enabling continuous antigen acquisition followed by efficient cross-presentation. In multiple solid tumor models, including melanoma and colorectal cancer, this integrated platform achieved tumor clearance rates ranging from 50% to 100% when combined with immune checkpoint inhibition, significantly outperforming either DC therapy or nanoparticle vaccination alone. These results emphasize that DC-based living adjuvants can be designed to function as dynamic and adaptive components within an evolving tumor antigen landscape, rather than as static antigen carriers [99]. Apart from *in vivo* antigen loading, immunomodulatory agents can also be installed on adoptively transferred dendritic cells *in vivo via* metabolic glycan labeling (Fig. 3B). Han et al. reported that dendritic cells cultured with tetraacetyl-N-azidoacetylmannosamine (Ac4ManNAz) (DC-N_3_) showed enhanced DC-vaccine activity, attributed to the reduced mobility of membrane proteins and increased expression of costimulatory molecules [84]. As a result, DC-N3 itself can already strengthen the tumor control in the therapeutic model against the murine E.G7-OVA tumor (Fig. 3C). Notably, surface azide groups persisted for at least 72 h, creating a practical window to conjugate exogenous alkyne-functionalized cytokines (*e.g.*, DBCO-IL-15) through bioorthogonal click chemistry. In a therapeutic E.G7-OVA model, DC-N3 alone yielded 1/7 long-term survivors at day 200 post-inoculation; DC-N3 plus DBCO-IL-15 improved survival to 3/7, whereas DC-N3 plus soluble IL-15 failed to confer durable benefit, with all mice succumbing before day 100 (Fig. 3D).

Taken together, these studies reveal a coherent trajectory in the evolution of DC-based cancer vaccination, systematically addressing challenges ranging from lymphoid organ delivery efficiency and immune synapse potency to intracellular programming and dynamic antigen handling. Across these diverse bioengineering strategies, the unifying principle is the use of dendritic cells as endogenous living adjuvants that integrate antigen delivery with immune activation through their intrinsic biology, while remaining amenable to precise chemical and material engineering. This shift from DCs as passive vaccine vehicles to active, programmable immune modulators provides a mechanistic and translational rationale for revisiting DC-based vaccination within a living adjuvant framework.

### 3.2 B cells

B cells are traditionally recognized for their role in antibody production, yet they also serve as efficient antigen-presenting cells for cognate antigens and coordinate T cell help in secondary lymphoid organs [[100], [101], [102]]. *In vivo*, B cells continuously acquire antigens through multiple mechanisms, including B cell receptor (BCR)-mediated internalization of cognate antigen, complement receptor-mediated uptake of opsonized antigens or immune complexes *via* CR2 (CD21) and CR1 (CD35) [[102], [103], [104]]. These uptake pathways are tightly linked to intracellular processing and presentation, predominantly on MHC class II, enabling B cells to engage CD4^+^ T cells and support T follicular helper-dependent follicular responses [105]. Under appropriate activation conditions, B cells upregulate costimulatory molecules such as CD80 and CD86 and secrete cytokines that modulate T cell differentiation [106,107]. In parallel, adoptively transferred B cells can recirculate and home to the spleen and lymph nodes, where they localize to follicular and white-pulp microanatomical niches [[108], [109], [110], [111]]. Together, these features position B cells as endogenous carriers that can deliver antigenic or immunomodulatory signals into lymphoid tissue while maintaining prolonged and repeated interactions with other immune cells.

**Fig. 4:**
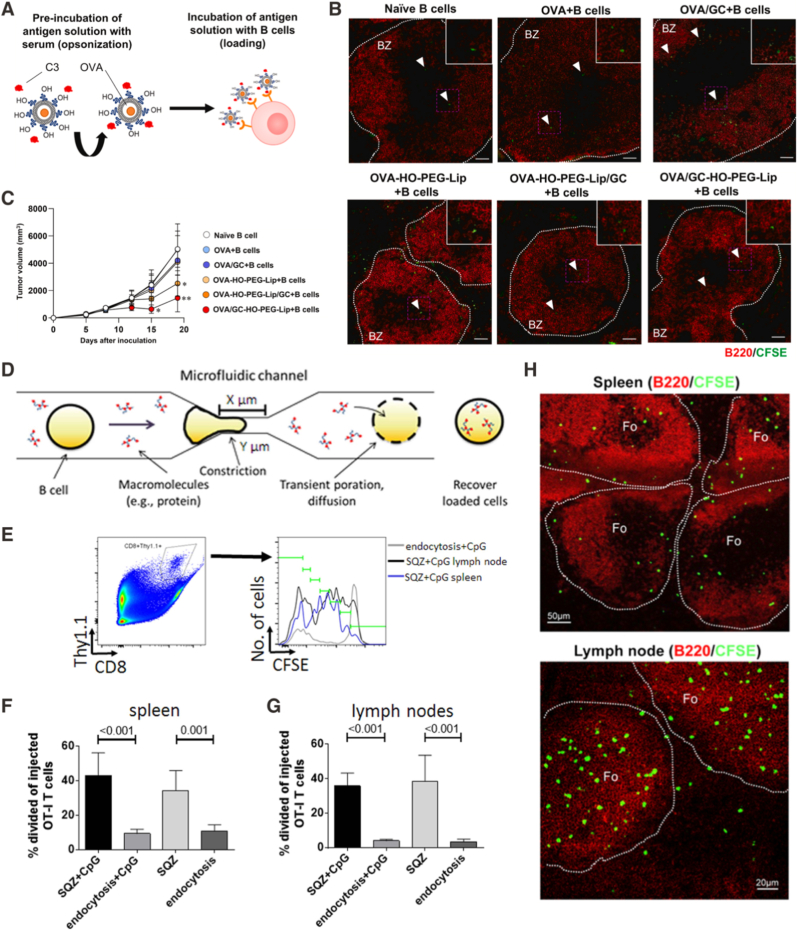
**Engineering strategies for B cell-based living adjuvants in cancer vaccination. (A)***Ex vivo* loading of B cells with antigen and immunostimulatory agents through nanoparticle-mediated delivery before adoptive transfer. **(B)** Biodistribution of transferred B cells in the spleen; CFSE-labeled B cells are shown in green. **(C)** Antitumor efficacy of adoptively transferred B cells prepared with different vaccine formulations in the E.G7-OVA model [113]. Copyright 2025, *Elsevier B.V.***(D)** Microfluidic “squeeze” (mechanoporation) platform for cytosolic antigen loading into B cells *ex vivo*. **(E)** Flow cytometry gating strategy for adoptively transferred OT-I CD8 T cells. **(F**–**G)** Proliferation of OT-I CD8 T cells in the spleen **(F)** and lymph nodes **(G)** across treatment groups, quantified by division tracking [114]. Copyright 2015, Springer Nature, *The Author(s).***(H)** Representative images showing B cell localization in lymph nodes and spleen at 24 h post-injection [115]. Copyright 2013, Elsevier, *American Society of Hematology.* (For interpretation of the references to color in this figure legend, the reader is referred to the Web version of this article.)

From an engineering perspective, the major limitation of B cells compared with dendritic cells may be their relatively low efficiency of nonspecific antigen uptake [102,112]. As a result, early work focused on improving the route through which antigens are loaded into B cells before transfusion. Kawaguchi et al. reported an *in vitro* nanocarrier-based antigen-loading system designed to enhance antigen uptake with primary B cells [113]. In this study, antigens and an immunostimulatory glycolipid (GC) were co-encapsulated within PEG-modified liposomes and further opsonized *ex vivo* in serum to promote B cell loading *ex vivo* (Fig. 4A). Antigen-loaded B cells were subsequently transfused into tumor-bearing mice, where they could accumulate in the spleen (Fig. 4B). In an E.G7-OVA thymoma model, animals receiving antigen-loaded B cells exhibited significant suppression of tumor growth compared with mice receiving unloaded B cells or free antigen controls (Fig. 4C). And they observed an elevated OVA-specific T cell response and anti-OVA IgG antibody concentration in the serum. While nanocarrier-assisted loading increases the quantity of antigen delivered, it does not by itself control intracellular routing steps that govern MHC-I antigen processing and cross-priming [116]. To overcome this constraint, Szeto et al. reported a microfluidic mechano-poration strategy that enables direct cytosolic delivery of protein antigens into live B cells [114]. In this system, B cells were passed through a microfluidic device that transiently disrupted the plasma membrane, allowing diffusion of soluble antigen into the cytosol without reliance on receptor-mediated endocytosis (Fig. 4D). The design rationale was that cytosolic access would favor proteasome and TAP-dependent MHC-I loading, thereby enhancing antigen-specific CD8 T-cell priming [50]. Following adoptive transfer, these engineered B cells induced robust proliferation of antigen-specific OT-I CD8 T cells in both spleen and lymph nodes. Quantitatively, antigen-loaded squeezed (SQZ) B cells elicited approximately ∼45% (CpG-activated) or ∼35% (resting) division of injected OT-I cells in spleen and ∼40% (CpG-activated) or ∼35% (resting) in lymph nodes, compared with ∼4% baseline division for endocytosis controls in lymph nodes (Fig. 4E–G).

Beyond antigen loading and intracellular processing, the microanatomical context of antigen delivery can set the ceiling for recall expansion and durability during booster immunization. Zhang et al. reported that B cell delivery of viral-vectored vaccines can bypass effector CD8^+^ T cell-mediated negative feedback during boosting [115]. They showed that pre-existing effector T cells rapidly eliminate antigen-bearing APCs during repeated vaccination, preventing timely engagement and expansion of memory CD8 T cells. To circumvent this barrier, viral vectors were first associated with B cells *ex vivo* before administration. After transfer, the vector-loaded B cells could accumulate in follicular regions of secondary lymphoid organs that are anatomically separated from effector T cells and positioned close to memory CD8 T cells (Fig. 4H). In this setting, B cells did not function as the principal APCs. Instead, classic CD11c^+^ dendritic cells drove the secondary CD8 T cell response. Consistently, B cell-mediated vector delivery enhanced peptide-specific CD8 T cells compared with DC-mediated delivery *in vivo*.

Expanding beyond physical delivery, emerging evidence highlights the capacity of B cells themselves to function as immune programming partners in tumor settings. Li et al. provided a cellular-adjuvant paradigm in which tumor-reactive B cells amplify endogenous T-cell immunity by rewiring how tumor antigens are handled *in vivo* [117]. They isolated B cells from tumor-draining lymph nodes of 4T1-bearing mice and licensed them *ex vivo* with CD40 stimulation plus LPS before adoptive transfer. The transferred B cells reduced primary tumor burden and suppressed spontaneous lung metastasis, and they further potentiated tumor regression when combined with activated T cells. Mechanistically, these B cells generated tumor-reactive immunoglobulins and antibody-dependent effector activity. From an adjuvant perspective, the antibody response could opsonize tumor-derived antigens and form immune complexes, which can be preferentially internalized by Fc receptor-expressing antigen-presenting cells and enhance antigen presentation (including cross-presentation), thereby amplifying endogenous CD8^+^ T-cell recall or effector responses [118]. Consistent with this programming partner role, the therapy was accompanied by strengthened host CD8^+^ responses with enhanced IFN-*γ* production and cytolytic function. Together, this study illustrates how B cells can behave as living adjuvants: not by carrying antigens or providing immunostimulatory signals, but by coordinating antigen opsonization, APC-accessible antigen formatting, and the amplification of T cell responses within the host.

Taken together, these studies outline a coherent engineering trajectory for B-cell-based living adjuvants. Initial efforts addressed the fundamental challenge of antigen entry, using nanocarriers or physical delivery to increase loading and control intracellular antigen processing routes. Subsequent work leveraged the natural homing and microanatomical positioning of B cells to deliver antigens or vectors into lymphoid niches that favor durable immune responses. More recent studies have shifted attention toward the capacity of B cells to actively program immune function through direct cellular interactions, antibody-mediated effects, and transient activation states. Across these implementations, the defining feature is not a single molecular adjuvant, but the use of intact, self-derived B cells as living platforms that integrate delivery, spatial organization, and immune instruction. This integration provides a mechanistic rationale for considering B cells as endogenous living adjuvants within cancer vaccination strategies.

### 3.3 Red blood cell

Red blood cells (RBCs) are the most abundant and long-lived cellular component in the circulation and are optimized for systemic transport and deformability [119,120]. In humans, RBCs circulate for ∼120 days and repeatedly traverse immune-filtering organs, particularly the spleen and liver, where senescent or mechanically compromised RBCs are removed by specialized macrophage populations and their iron is recycled. Although RBCs are not professional antigen-presenting cells, their predictable clearance through the mononuclear phagocyte system provides a clinically familiar trafficking route that can be leveraged for delivery, such that cargos adsorbed onto or coupled to intact RBCs can exhibit altered organ distribution and enhanced access to macrophage-rich compartments, depending on the engineering strategy [121,122]. RBCs can be modified *ex vivo* or *in situ* through adsorption, chemical coupling, or encapsulation approaches while retaining a self-derived cellular chassis, which supports the use of autologous RBCs as a delivery platform in vaccine and immunotherapy design [67,123].

Early work established that erythrocyte-based antigen delivery itself constitutes a powerful routing signal into immune processing pathways, but that the resulting immune outcome depends critically on the accompanying context. Kontos et al. reported an approach in which antigens were engineered for *in situ* binding to endogenous erythrocytes following intravenous administration [124]. In this system, erythrocyte binding redirected antigen to splenic and hepatic immune cell populations, resulting in antigen exposure that preferentially drove deletion of antigen-specific CD4 and CD8 T cells and systemic tolerance rather than productive immunity. This study demonstrated that erythrocyte hitchhiking can reliably enforce immune exposure, yet in the absence of activating signals, it can be biased toward tolerance. Although not designed as a cancer vaccine, this work established an essential design principle for erythrocyte-based platforms and provides a mechanistic baseline for following activating strategies.

**Fig. 5:**
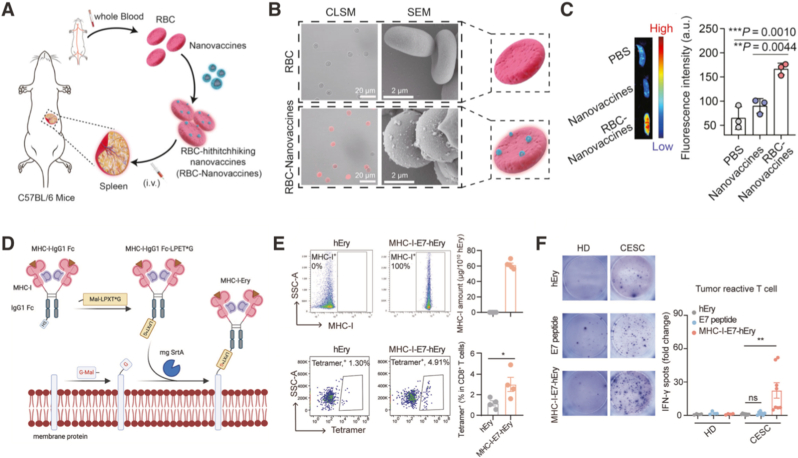
**Engineering strategies for red blood cell-based living adjuvants in cancer vaccination. (A)** Fabrication workflow for an RBC-hitchhiking nanovaccine designed to target the spleen for cancer immunotherapy. **(B)** Confocal microscopy (left) and SEM (right) images of untreated RBCs and RBCs decorated with DiI-labeled nanovaccines at a nanovaccine-to-RBC ratio of 100:1. **(C)** IVIS images and corresponding quantification of splenic signal 48 h after intravenous injection of free DNA nanovaccines *versus* RBC-hitchhiking DNA nanovaccines [125]. Copyright 2023, Springer Nature, *The Authors.***(D)** Schematic of MHC-I-Ery preparation. G-Mal denotes GAASK-6-maleimide; Mal-LPET∗G contains a 2-hydroxyacetic acid linker (asterisk) between threonine and glycine. **(E)** Left, flow cytometry of MHC-I-E7-hEry stained with anti-human IgG; right, ELISA-based quantification of MHC-I-E7 density on erythrocytes. **(F)** IFN-γ ELISpot analysis of antigen-specific T cell responses: PBMCs from HPV16-positive cervical squamous cell carcinoma (CESC) patients or healthy donors were co-cultured for 48 h with hEry, MHC-I-E7-hEry, or the HPV16 E7 peptide [126]. Copyright 2024, Springer Nature, *The Authors.* (For interpretation of the references to color in this figure legend, the reader is referred to the Web version of this article.)

Building on erythrocyte-mediated routing, Wu et al. developed a spleen-targeted personalized neoantigen DNA nanovaccine by deliberately hitchhiking polymer-lipid (PEI_25000_-C_14_, TLR4 agonist) nanoparticles encapsulating neoantigen-carrying plasmid DNA onto pre-isolated red blood cells for intravenous administration (Fig. 5A and B) [125]. In this design, erythrocyte-mediated delivery acted as a deterministic trafficking cue that preferentially distributed the vaccine in the spleen, thereby increasing neoantigen expression within antigen-presenting cells and eliciting markedly stronger neoantigen-specific T-cell immunity than the non-hitchhiked nanovaccine (Fig. 5C). Functionally, the regimen slowed aggressive HCC growth. When paired with anti-PD-1 checkpoint blockade, this RBC-based splenic vaccine achieved complete tumor regression with high response rates (including 75% in subcutaneous and 100% in orthotopic models) in murine Hepa1-6 tumor models. A complementary biomimetic strategy was reported by Ukidve et al., who described erythrocyte-driven immunization (EDIT) by enabling nanoparticle vaccines to hitchhike on red blood cells for a spleen-targeted cancer vaccine [127]. In this system, the vaccine nanoparticles themselves act as potent adjuvants. Their particulate nature and high-density antigen display significantly upregulate costimulatory markers (CD80) on dendritic cells, comparable to the effect of LPS. And the adsorption of nanoparticles induces a controlled, moderate upregulation of phosphatidylserine on the RBC surface. Unlike the high phosphatidylserine levels that trigger suicidal clearance, this intermediate exposure serves as a pro-interaction signal, acting as a molecular glue that facilitates docking with splenic dendritic cells. The dense coating of nanoparticles provides physical masking of CD47 (the “don't-eat-me” signal). Crucially, the mechanistic basis for this immune potentiation was further validated through a macrophage depletion assay using clodronate liposomes. They discovered that splenic cargo uptake remained robust post-depletion, which indicated that the antigens on RBCs are selectively transferred to professional APCs rather than being sequestered during the silent, terminal degradation typical of red-pulp macrophages. Notably, this immune potentiation was achieved with minimal systemic inflammation while achieving comparable tumor inhibition capability as the antigen + CpG group, highlighting erythrocytes as living delivery partners that couple systemic transport with predictable handoff to immune-processing niches.

Mechanistically, the stark contrast between erythrocyte-induced tolerance and productive immunity highlights a critical immunological switch governed by the synergistic integration of innate signals and the specific APC subsets engaged. In the steady state, the non-inflammatory clearance of senescent RBCs lacks co-stimulation, leading to T-cell deletion or anergy. To flip this switch toward immunity, engineering strategies must provide Signal 2 (co-stimulation) and Signal 3 (cytokines). This is achieved either by co-loading RBCs with potent innate agonists or, as exemplified by the EDIT platform, by leveraging the intrinsic adjuvant properties of the cargo (nanoparticle) itself. In the latter case, the particulate nature and high-density antigen display of the nanoparticles are potentially sufficient to trigger DC maturation. However, the RBC carrier provides the decisive routing-to-activation cue: by modulating surface markers such as phosphatidylserine and CD47, these platforms ensure a selective cargo hand-off to cross-presenting splenic DCs rather than terminal, silent degradation in red-pulp macrophages.

In addition to using erythrocytes as routing carriers for antigenic cargo, their surfaces can be engineered to display immunologically defined ligands, thereby converting predictable physiological trafficking into a more instructive, signal-presenting interface. Liu et al. reported an erythrocyte-MHC-I conjugate for cancer treatment, in which peptide-MHC-I complexes were covalently coupled onto intact erythrocytes and support peptide-specific CD8^+^ T-cell expansion (Fig. 5D and E) [126]. An *ex vivo* PBMC co-culture assay showed that MHC-I–E7-conjugated human erythrocytes induced the strongest antigen-specific response in PBMCs from HPV16-positive cervical squamous cell carcinoma (CESC) patients (Fig. 5F). In murine subcutaneous MC38-HPV16 tumor models, treatment with erythrocyte-MHC-I conjugates significantly reduced tumor burden (∼71.6% tumor-volume decrease *versus* controls) and increased antigen-specific cytotoxic T-cell responses. These data support erythrocytes as a functional living adjuvant platform, in which the RBC carrier enhances immune engagement while the displayed MHC-I-E7 complex confers HPV16 E7 specificity in a clinically relevant HPV-associated cancer setting. Combination with immune checkpoint blockade further enhanced efficacy, yielding complete remissions in a substantial fraction of animals (∼40% with anti-PD-1 in the reported setting). Importantly, matched soluble peptide-MHC-I complex at comparable doses failed to recapitulate these effects, underscoring that erythrocyte display and physiological trafficking were critical determinants of therapeutic output. In interpreting these results, several mechanistic and translational caveats remain. First, although MHC-I-decorated erythrocytes clearly required the presence of accessory immune cells to activate CD8 T cells, the identity of the co-stimulatory circuit was not definitively established. Second, the choice of HPV16 E6/E7 epitopes represents a highly immunogenic, pathogen-derived tumor antigen context, which likely benefits from pre-existing antigen-experienced T-cell repertoires and reduced tolerance constraints. Accordingly, the extent to which erythrocytes display alone can drive *de novo* priming, or sustain responses against weak self-derived tumor antigens in poorly inflamed solid tumors, remains to be demonstrated.

While spleen-focused priming represents one major application, erythrocyte-based living adjuvants can also be harnessed to initiate *in situ* vaccination efficacy. Zhao et al. reported systemic tumor suppression *via* preferential accumulation of erythrocyte-anchored chemokine-encapsulating nanoparticles in lung metastases [128]. In this system, chemokine-loaded nanoparticles were non-covalently anchored to erythrocyte surfaces before intravenous administration. Because erythrocytes naturally perfuse and transiently traffic through pulmonary microvasculature, the anchored chemokine-loaded nanoparticles could detach from the RBC carrier and deposit in lung microvasculature, generating localized immune cell recruitment within metastatic lung lesions. In multiple lung metastasis models, this approach resulted in significant suppression of metastatic progression and prolonged survival compared with control treatments. Notably, treatment also induced systemic immune memory capable of suppressing distant tumor growth upon rechallenge, indicating that erythrocyte-mediated local immune education can indeed propagate into the *in situ* vaccination process.

These studies showcase the development of erythrocyte-based vaccine engineering. Initial demonstrations established erythrocyte hitchhiking as a reliable route for efficient immune exposure. Subsequent designs leveraged this routing to enhance splenic priming. And more recent platforms transformed erythrocytes into activating or instructive immune units through controlled presentation of antigens or innate cues. Across these implementations, the defining feature is the use of intact, self-derived red blood cells as endogenous living adjuvants that integrate systemic delivery with immune modulation through physiological circulation and clearance pathways. This integration provides a strong mechanistic and translational rationale for including erythrocyte-based platforms within the broader framework of living adjuvants for cancer vaccination.

### 3.4 Mesenchymal stromal cell

Mesenchymal stromal cells are not professional antigen-presenting cells, as they typically exhibit low basal expression of MHC-II and canonical costimulatory ligands (*e.g.*, CD80/CD86 and CD40), yet they possess a combination of biological properties that make them uniquely amenable to engineering as endogenous living adjuvants [129]. MSCs can be readily isolated and expanded *ex vivo*, maintain viability after systemic or local administration, and have been reported to traffic to and persist within injury tissues, with context-dependent localization in tumor-associated microenvironments [130]. In parallel, MSCs engage extensively with immune cells through both secreted factors and contact-dependent mechanisms. Under physiological conditions, this interaction profile often results in immunoregulatory or suppressive effects, which have motivated the clinical exploration of MSCs in inflammatory and autoimmune indications and, more prominently, acute graft-versus-host disease [[131], [132], [133]]. For cancer vaccination, however, the same biology presents an opportunity rather than an obstacle, as MSC-mediated immune modulation can be reprogrammed by engineering them to alter their identity, location, and duration of the signals delivered, thereby coupling tissue-level delivery with immune modulation while relying on endogenous antigen-presenting and myeloid compartments to supply complete T-cell priming signals. Together, MSCs have emerged as a cellular platform in which delivery and immune instruction can be integrated within a single viable, host-derived system [134].

Early efforts to exploit MSCs in cancer immunotherapy focused on using MSCs as a vehicle to deliver immunomodulatory cues to tumors, motivated by the limitations of systemic administration of immunomodulatory proteins (including toxicities and insufficient tumor infiltration). Yin et al. engineered adipose tissue-derived MSCs to express CXCL9 and OX40L, and reported that these modified MSCs migrated to and resided in tumors following systemic administration [135]. In multiple syngeneic tumor models, treatment was associated with increased intratumoral CD8^+^ T cells and NK cells and increased expression of antitumor cytokines and cytolytic proteins, alongside inhibition of tumor growth and metastasis. In an AOM/DSS-induced colorectal cancer model, the same MSC-based delivery of CXCL9 and OX40L reduced colorectal tumor numbers. Although this strategy does not deliver a defined tumor antigen in the way a classical vaccine does, it can be viewed as vaccine-like because it promotes systemic expansion and functional activation of endogenous cytotoxic lymphocytes while conditioning the tumor site to support priming and amplification rather than tolerance.

While tumor-localized immune activation can improve effector cell access, durable antitumor immunity also requires antigen-specific priming. Abusarah et al. engineered MSCs to stably express the immunoproteasome, converting them into adjuvant-like platforms with enhanced expression of MHC-I and CD80, and increased chemokine and IL-12 secretion, thereby supporting cross-presentation and T cell priming [136]. In established E.G7 lymphoma, vaccination delayed tumor growth for more than 6 weeks, but immune pressure promoted antigen loss. Using tumor lysates and immune modulation improved outcomes: in the EL4 murine model, vaccination with engineered MSCs yielded ∼30% survival, increasing to ∼40% with 4-1BB agonism and up to ∼80% with checkpoint blockade at 7 weeks after tumor inoculation.

In addition to stable genetic modification, transient programming strategies have been explored to balance immunostimulatory potency with safety and controllability. Cantero et al. reported the use of *in vitro* transduced mRNA to transiently express GM-CSF in MSCs, generating a short-lived but potent source of myeloid differentiation signals within tumor tissue [137]. Engineered MSCs secreted functional GM-CSF that promoted dendritic cell differentiation and inflammatory macrophage polarization *in vitro*. *In vivo*, peritumoral administration of GM-CSF-expressing MSCs reduced tumor growth in immunocompetent gastrointestinal cancer models. When combined with low-dose doxorubicin to induce immunogenic cell death and increase endogenous antigen availability, the MSC-based immune conditioning strategy significantly extended survival in a hepatocellular carcinoma model. Median survival increased from approximately 15 days in control animals to 27.5 days in the combination group, with a subset of animals showing prolonged survival beyond 30 days. This work demonstrates how MSCs can function as transient immune conditioning units that support *in situ* vaccination rather than as direct antigen carriers.

Collectively, these studies support a view of MSCs as endogenous living adjuvants whose function emerges from the integration of localization, immune modulation, and engineering modality. MSC-based platforms have progressed from delivering localized chemotactic and costimulatory cues to participating directly in antigen processing, to acting as transient immune conditioning units that amplify endogenous antigen release. Across these implementations, therapeutic performance is determined not by a single engineered factor but by the coordination of antigen availability, immune activation state, and spatial distribution.

### 3.5 Tumor cell platforms

Tumor cell-based vaccination strategies have historically been developed to provide comprehensive antigen repertoires, but antigen breadth alone does not define adjuvant function. In most classical whole tumor cell vaccines, tumor cells are irradiated or otherwise inactivated before administration, and immune activation arises primarily from immunogenic cell death and passive antigen release [138,139]. Although these inactivated platforms represent borderline cases that fall outside our strict definition of endogenous living adjuvants (as established in the Introduction, due to loss of cellular viability and active biological participation), they nevertheless expand the conceptual and design framework in important ways: they illustrate the evolutionary path from passive antigen sources toward active adjuvant competence, preserve trafficking features that can inform living cell engineering, and highlight translational advantages in manufacturability and safety. In this section, we therefore do not attempt to comprehensively summarize whole tumor cell vaccines. Instead, we focus on tumor cell-based designs in which adjuvant function is explicitly engineered, meaning that tumor cells are used to actively shape innate immune activation, antigen handoff to professional antigen-presenting cells, or the spatial organization of immune priming.

One of the earliest and most instructive approaches to endow tumor cell vaccines with adjuvant properties was the introduction of cytokine-driven immune recruitment at the vaccination site. Dranoff et al. reported tumor cells genetically modified to secrete GM-CSF before irradiation and administration [140]. The design rationale was to transform the injection site into a localized immune-activating niche by recruiting and differentiating dendritic cells and other myeloid populations, thereby enhancing cross presentation of tumor antigens by host APCs. In preclinical tumor models, GM-CSF-secreting tumor cell vaccines enhanced infiltration of dendritic cells and lymphocytes at vaccination sites and were associated with immune-mediated tumor regression in a subset of subjects. Belagenpumatucel-L (Lucanix) exemplifies a tumor-cell vaccine strategy that attempts to relieve tumor-derived immunosuppression to create a more permissive, adjuvant-like vaccination milieu. The formulation consists of irradiated allogeneic NSCLC cell lines engineered with a TGF-*β*2 antisense construct to reduce the production of this immunosuppressive cytokine during vaccination [141,142]. This design was intended to dismantle a dominant inhibitory cue and thereby facilitate host APC uptake and priming against a broad tumor-antigen repertoire carried by the vaccine cells. While late-stage clinical benefit was limited, immune monitoring in early studies reported measurable immunologic perturbations (*e.g.*, altered cytokine responses and evidence of vaccine-induced immune reactivity), supporting the concept that tumor-cell platforms can be rationally modified to shift from passive antigen sources toward adjuvant-competent vaccine substrates.

**Fig. 6:**
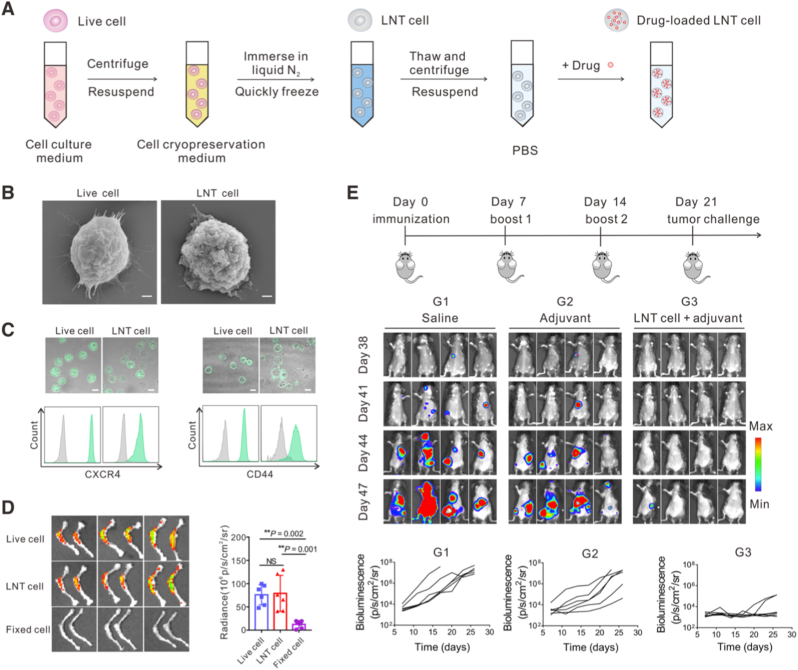
**Engineering strategies for tumor cell-based adjuvants in cancer vaccination. (A)** Schematic of the fabrication workflow for liquid nitrogen-treated tumor cells (LNT). **(B)** SEM images of live tumor cells and LNT cells. **(C)** CXCR4 and CD44 expression on live and LNT C1498 cells analyzed by confocal microscopy (top) and flow cytometry (bottom). **(D)** Representative fluorescence images and quantitative signal intensity of femur (bone) collected 6 h after intravenous injection of Cy5.5-labeled live C1498 cells, LNT C1498 cells, or paraformaldehyde-fixed C1498 cells. **(E)** Vaccination and challenge scheme. Bioluminescence images and quantification in mice pre-immunized with different formulations [143]. Copyright 2020, *The American Association for the Advancement of Science*.

A boundary case that further clarifies the distinction between antigen source and adjuvant function is the use of inactivated tumor cells as functional delivery vehicles. Ci et al. reported a liquid nitrogen cryo-shocking method that converts live cancer cells into dead but structurally preserved cells termed LNT cells (Fig. 6A and B) [143]. These inactivated cells retained homing-associated surface proteins such as CXCR4 and CD44 (Fig. 6C), enabling preferential accumulation in bone marrow following intravenous administration (Fig. 6D). Beyond these specific chemokine axes, tumor-derived cells often exhibit a distinctive homotypic targeting propensity. Current empirical evidence for this phenomenon stems primarily from macro-scale biodistribution studies, such as IVIS imaging, which demonstrate that adoptively transferred tumor cells could enrich in syngeneic tumor tissues [144]. While the precise molecular orchestration of this self-recognition is not yet fully parsed, the observed selectivity provides a strategic navigation system for site-specific delivery. In a C1498 acute myeloid leukemia model, LNT cells loaded with doxorubicin achieved superior leukemia control compared with free drug. When combined with an innate immune agonist, LNT cell-based treatment also induced durable protective immunity upon challenge (Fig. 6E). While this platform demonstrates that tumor-derived cells can preserve tumor tissue targeting and support immune activation after inactivation, the absence of biological viability places such systems outside the strict definition of living adjuvants. We therefore use these studies to illustrate principles of tissue-directed immune programming for *in situ* vaccination, while reserving the term living adjuvant for platforms in which cellular activity is retained [145,146]. Looking ahead, the inherent homing fidelity of tumor cells, even if mechanistically incomplete in our current understanding, offers a roadmap for engineering next-generation endogenous living adjuvants. Taking advantage of this ability, future platforms could utilize viable but proliferation-controlled tumor cells as “Trojan Horses” to coordinate immune priming spatially. These engineered cells could be designed to sense the tumor microenvironment and locally release cytokines or checkpoint inhibitors specifically within metastatic niches. By transforming homing-capable tumor cells into localized bio-factories, researchers can effectively initiate immune recruitment in cold tumors while minimizing systemic inflammatory toxicities. Taken together, tumor cell platforms reveal that adjuvant function can be introduced through multiple mechanisms, including local cytokine-mediated immune recruitment and removal of immunosuppressive signaling. The logical progression across these examples highlights that tumor cells do not inherently function as adjuvants, but can acquire adjuvant properties when engineered to control how antigens are perceived by the immune system.

## 4 Comparative perspective and design principles of endogenous living adjuvant

Endogenous living adjuvants differ from conventional adjuvants because the carrier itself determines which antigen-presenting cells encounter the antigen, whether the antigen enters the cross-presentation pathway, and how long peptide-MHC I complexes persist. Conventional adjuvants such as alum, CpG, or MPLA primarily provide innate activation signals that increase antigen uptake and drive APC maturation (*e.g.*, upregulation of CD80, CD86, and IL-12 production), yet their *in vivo* impact is constrained by local dispersion, lymphatic drainage, and clearance, which limits the magnitude and duration of antigen availability for T cell priming [17,18]. By contrast, living platforms add a second set of determinants: (1) cellular trafficking routes, (2) carrier-APC contact features, and (3) controllable persistence. Therefore, the immune outcomes depend not only on the cargo delivered (antigen plus innate agonists or cytokines), but also on which carrier brings the cargo to which anatomical immunological compartment and which APC subset (cDC1 *versus* macrophages/monocytes) actually processes and presents it.

A key advantage of endogenous living adjuvants is that the priming compartment and the dominant antigen-processing route can be selected by carriers’ inherent biology. Conventional adjuvants tend to initiate responses at the injection site and draining lymph nodes, where resident and recruited APCs sample soluble or particulate cues [17,147]. Living systems can be designed to bias exposure toward compartments and APC subsets that are harder to access with static materials. Adoptively transferred dendritic cells can deliver pre-formed antigen-MHC complexes and high CD80 and CD86 directly to T cell zones, supporting efficient naive CD8 T cell activation and early effector differentiation [148]. B cell-based carriers preferentially access follicles and can retain antigen on the surface and recycle it for repeated presentation to Tfh cells, thereby amplifying germinal center reactions and antibody maturation when the goal is humoral immunity [149]. Red blood cell platforms exploit physiological splenic clearance: erythrocyte-associated antigens and ligands can be sampled during erythrophagocytosis by splenic macrophages and DCs, converting routine turnover into recurrent antigen exposure while keeping systemic cytokines at a relatively low level [150]. Mesenchymal stromal cells often accumulate in inflamed tissues and many tumors, increasing the probability that antigens, chemokines, and costimulatory cues are encountered by APCs within the tumor microenvironment, where antigen release is ongoing [151]. Tumor cell-based platforms illustrate the converse: antigen supply alone can be non-productive if uptake is dominated by non-inflammatory phagocytosis, whereas adding strong innate cues (*e.g.*, type I interferon-inducing signals) can shift processing toward DC maturation, cross-presentation, and subsequent priming in draining lymph nodes [143]. In endogenous living adjuvant design, the central question becomes which immune compartment receives antigen first, which APC subset dominates processing, and whether cross-presentation is favored over rapid lysosomal degradation.

Endogenous living adjuvants also reshape immune stimulation by concentrating signals at defined cell-cell interfaces [152]. Conventional small molecule-based adjuvants activate innate sensors effectively, but they generally act through diffuse exposure of local phagocytes and cytokine networks, which becomes difficult to confine at higher doses or after systemic delivery [153]. Living platforms can localize the same signal classes: costimulatory ligands (CD80/CD86, 4-1BBL), chemokines (CXCL9/10, CCL19/21), and innate agonists that trigger type I interferon or NF-κB pathways *via* cell-bound presentation or contact-dependent transfer, so that activation is biased toward APCs and lymphocytes physically interacting with the carrier [154]. For DC-based platforms, this means restricting antigen presentation and costimulation to the immediate T cell contact zone [155]. For RBC-based platforms, it means that multivalent ligands are interpreted during professional phagocyte processing in the spleen rather than requiring high soluble concentrations [126]. For MSC-based platforms, it means that chemokine and costimulatory programs can be enriched at tumor or peritumoral sites, rather than generating generalized inflammation [156]. Across these systems, the practical advantage is the higher local signal density at the relevant cellular synapse with less systemic exposure.

Temporal control is a second axis in which living carriers offer capabilities difficult to reproduce with conventional formulations [157]. Many molecular adjuvants produce a short inflammatory pulse whose duration is governed by pharmacokinetics and negative immune feedback [158,159]. Living carriers introduce persistence that can be tuned through carrier lifespan and clearance pathways, thereby shaping the duration of antigen presentation, costimulation, and cytokine support. This persistence is not inherently beneficial: overextended stimulation can drive T cell dysfunction or exhaustion, whereas brief exposure may fail to establish memory [[160], [161], [162]]. DC transfer typically yields a defined window of presentation aligned with early priming kinetics [163,164]. RBC platforms generate repeated exposure through turnover yet remain time-limited by physiological clearance [165]. MSC platforms may persist longer in inflamed milieus and therefore require control to avoid context-dependent shifts toward immunoregulatory phenotypes [166]. Effective and desired endogenous living adjuvant design, therefore, treats persistence as a matched parameter to antigen release kinetics and T cell differentiation timelines, not as a goal to maximize. To further aid readers in selecting an appropriate endogenous living adjuvant platform for specific cancer vaccination goals, we provide a comparative synthesis across the five cellular classes discussed (Table 1).

**Table 1:** Comparative overview of endogenous living adjuvant platforms for cancer vaccination.

**Platform**	**Homing and Priming Site**	**Mechanism and CD8^+^ Priming Route**	**Persistence and Dosing**	**Manufacturing and Engineering Complexity**	**Key Limitations and Safety**	**Overall Suitability**
**DCs**	dLN, Spleen	Direct presentation. CD8 Route: Direct MHC-I presentation by transferred cDC1s or engineered cross-priming.	Transient (days); Fast-acting; Single or prime-boost.	Autologous. Moderate complexity (*ex vivo* maturation/loading); Low scalability.	Poor lymph node homing efficiency; Susceptibility to TME suppression; Highly well-tolerated (mild local reactions).	High-fidelity priming for defined-antigen vaccines.
**B Cells**	Spleen, dLN	BCR-mediated endocytosis. CD8 Route: Antigen relay to host DCs for cross-priming	Moderate (weeks); Supports recall/memory expansion.	Autologous. High complexity (needs cytosolic delivery for MHC-I); Mod scalability.	Autoantibody risk; Suboptimal direct MHC-I loading and presentation.	Amplifying humoral and CD4^+^ help; Long-term memory.
**RBCs**	Spleen	Erythrophagocytosis. CD8 Route: Uptake of RBC-antigen conjugates by splenic APCs.	Long (up to 120d). Continuous/repeated splenic exposure.	Autologous/Allogeneic. Low complexity (surface conjugation); High scalability.	Hemolysis risk; Off-target liver sequestration.	Spleen-targeted delivery; Minimizing systemic toxicity.
**MSCs**	TME, Inflamed tissues	Paracrine conditioning. CD8 Route: Modulating TME to recruit and activate host DCs for *in situ* cross-priming.	Sustained (weeks); Context-dependent persistence.	Allogeneic (Off-the-shelf). Mod/High complexity (genetic circuits); High scalability.	Pro-tumorigenic shift; Off-target lung/liver trapping.	*In situ* vaccination; Reprogramming solid tumors.
**Tumor Cells**	Tumor, Injection site	ICD-mediated DAMP release. CD8 Route: Host DC phagocytosis of tumor debris plus innate signals.	Variable. Short if inactivated; Long if viable.	Autologous/Allogeneic. Mod complexity (cytokine gene insertion); High scalability.	Incomplete inactivation; Risk of promoting adaptive resistance.	Broad-antigen repertoire; *In situ* priming scaffolds.

In the future, other leukocyte lineages could extend the living-adjuvant concept as their recruitment kinetics and effector programs can be used to control when and where tumor antigens become available to cross-presenting dendritic cells. Neutrophils rapidly infiltrate inflamed tumors and readily take up particulate cargo. Importantly, antigen can be transferred to dendritic cells through uptake of neutrophil-associated substances, providing a possible time window that potentially can support CD8 T-cell priming *via* cross-presentation [167]. Beyond simple cargo transport, the mechanistic rationale for neutrophil-based adjuvants lies in their ability to provide potent endogenous immunomodulatory cues [168]. For instance, the release of neutrophil extracellular traps (NETs) and alarmin-rich granules can directly recruit and activate dendritic cells [[169], [170], [171]]. Specifically, NETs can serve as immunostimulatory physical scaffolds that concentrate antigens and alarmins, inducing a pro-inflammatory cytokine signature in dendritic cells [172]. Furthermore, granular peptides like cathelicidin LL-37 offer a concrete precedent for active programming. LL-37 has been shown to reprogram DCs toward an enhanced CD103^+^ cDC1-like phenotype by upregulating the transcription factor BATF3, thereby improving cross-presentation capacity and CCR7-dependent migration [170].

Eosinophils and basophils provide a distinct class. As innate sources of type-2 cytokines (notably IL-4/IL-13) and lipid and granule mediators, they can reprogram stromal and myeloid states and thereby influence whether intratumoral antigen release is routed into cross-presenting DC circuits *versus* phagocyte programs [173,174]. Eosinophils can actively shape the immune architecture by releasing eosinophil-derived neurotoxin (EDN), an endogenous alarmin that triggers TLR2 pathways to promote DC maturation [175]. Precedents also highlight their ability to secrete chemokines like CCL5 and CXCL10, which normalize tumor vasculature and facilitate robust CD8^+^ T-cell infiltration [176]. Similarly, basophils provide a unique amplification circuit. Upon activation by T cell-derived IL-3, they produce IL-4 that specifically rejuvenates cytotoxic T lymphocytes (CTLs), enhancing their persistence and IFN-γ production [177,178].

Mast cells, which are often positioned near vessels, can rapidly increase vascular permeability and shape leukocyte recruitment through the release of histamine, proteases, and cytokines, potentially changing which antigen-presenting cells gain access to antigen-rich sites and at what time [179]. The mechanistic value of mast cells as living adjuvants hinges on their role as vascular gatekeepers. It is well established that mast cell-derived mediators, such as TNF-*α* and CCL3, are indispensable for the rapid mobilization of DCs to lymph nodes [180,181]. By engineering the controlled degranulation of mast cells, future platforms could acutely increase vascular permeability to orchestrate the spatial infiltration of cDC1s into otherwise cold tumor niches. The opportunity is therefore not to place these lineages as professional antigen-presenting cells, but to engineer them as controllable immunomodulatory modules that tune APC access, APC composition, and the duration of an activating niche.

## 5 Translational considerations and future outlook

To systematically navigate the variables discussed in previous sections, we propose a four-step decision-flow to guide the rational design of endogenous living adjuvants. The first step is the selection of the cell source, which should be dictated by the intrinsic trafficking and lifespan of the cellular carrier. For instance, long-lived erythrocytes are ideal for sustained, spleen-targeted antigen presentation, whereas mesenchymal stromal cells or tumor cells are better suited for homing to inflamed or immunosuppressive tumor microenvironments. The second step involves choosing applicable engineering strategies based on the desired onset and duration of the signal. Surface conjugation (*e.g.*, lipid-insertion or click chemistry) offers rapid, defined signal presentation, whereas genetic circuits provide dynamic, environment-responsive cytokine release but require complex *ex vivo* manipulation. The third step is defining the delivery route, which must mechanically couple the carrier to the intended immune compartment. Intravenous delivery leverages systemic circulation for splenic or hepatic filtering, whereas subcutaneous or intradermal routes are required to drain efficiently into local lymph nodes. Finally, these choices culminate in targeting a specific immunological organ or niche. The design must align the carrier's destination with the right antigen-presenting cell subset, such as guiding cargos to splenic marginal zones to engage cross-presenting cDC1s, or directing them to the tumor interior to reprogram intratumoral dendritic cells. By following this decision-flow, researchers can rationally match the biological chassis to the specific spatial and temporal requirements of the target cancer.

However, conceptualizing an elegant platform on the drawing board is only the first hurdle. Translationally, endogenous living adjuvants should not be judged by whether they increase inflammation, because almost any classic adjuvant can do that. Their differentiator is that a viable (or cell-based) system imposes rules on where immune stimulation happens, which immune cells physically engage the cues, and how long those cues persist before being cleared [14,157,182,183]. The practical question for translation is therefore: can we convert these biological constraints into controllable product attributes, while keeping safety margins wider than the immune activation window?

To answer this, a first requirement is to define the product in a way that survives real manufacturing and regulatory review [184]. For endogenous living adjuvants, identity and purity are necessary but not sufficient. The platform must also carry a mechanism-linked potency assay that reflects its intended immune function rather than a generic viability readout [185]. For antigen-competent platforms, potency should be anchored to measurable steps in antigen handling and T cell priming, such as antigen uptake, cross-presentation capacity in a standardized model system, induction of APC maturation markers, and the ability to drive antigen-specific CD8 T-cell expansion *ex vivo*. For conditioning-type platforms that mainly reshape local immunity (for example, chemokine or costimulation delivery in tumors), potency should be tied to the expected directional changes in APC recruitment and activation, and effector T-cell entry, not just cytokine release. This matters because the central translational risk of endogenous living adjuvants is batch-to-batch drift: two MSC preparations may look similar by surface markers but behave differently in antigen processing, innate sensing, or immunoregulatory programs [186,187]. A potency assay that tracks the intended immune bottleneck is the most defensible way to lock the mechanism to release criteria.

Closely tied to product definition is the critical choice between personalized (autologous) and “off-the-shelf” (allogeneic) manufacturing. Autologous systems, such as the pioneering dendritic cell vaccine Sipuleucel-T, are highly biocompatible since they use the patient's own cells. However, they have historically struggled commercially due to complex logistics, high per-patient manufacturing costs, and the inconsistent quality of cells collected from heavily treated patients [73,188]. In contrast, allogeneic platforms offer a much more scalable and standardized approach, meaning the therapy is ready to use immediately. Yet, the main biological hurdle for these universal platforms is immune rejection (host-versus-graft responses). The patient's immune system might destroy the donor cells before they can effectively deliver their adjuvant signals. To solve this, future design principles must equip allogeneic cells with immune-evasion features. This can be achieved by selecting cell types that naturally avoid immune detection, like red blood cells or certain mesenchymal stromal cells, or by using gene editing to remove human leukocyte antigen (HLA) molecules, creating “universal” donor cells [150,189,190]. Moving from custom-made autologous processes to engineered, off-the-shelf products is a necessary step to make living adjuvants practical, affordable, and widely available.

Second, the anatomical variable that makes endogenous living adjuvants interesting must be translated into dosing logic, not left as a narrative flourish [14,157]. The route of administration is not merely a convenience choice. Rather, it defines which APC network will first interpret the signal, which lymphoid niche will amplify it, and which safety liabilities become dominant. Conventional adjuvants largely rely on a predictable injection-site to draining-lymph-node axis [191]. Living platforms can be positioned differently: dendritic cell and B cell approaches explicitly exploit secondary lymphoid organ traffic to engage naive T cells and follicular helper programs. Erythrocyte-based systems exploit repeated splenic processing during physiological clearance. MSC-based systems often accumulate in inflamed tissue and many tumor microenvironments, making them more suitable for conditioning immunity at disease sites rather than acting as classic depot adjuvants. Translationally, this means early clinical protocols should pre-specify a “distribution-to-effect” chain: a way to verify where the product goes (imaging, blood persistence, cell tracking), and a time-resolved set of biomarkers that match that distribution (for example, local APC activation and chemokine gradients if the goal is *in situ* priming). Without these readouts, endogenous living adjuvants risk being evaluated with endpoints designed for soluble adjuvants, which obscures what they uniquely contribute.

Third, durable benefit will often require building around predictable failure modes rather than assuming that stronger stimulation is always better [192,193]. Many endogenous living adjuvant platforms reveal a fundamental tension: once antigen-specific priming becomes effective, immune pressure can select for antigen loss or adaptive resistance [194]. This is not a minor detail. Rather, it is the mechanism by which “proof-of-principle” tumor regression fails to generalize. Therefore, antigen breadth and resistance management should be treated as design variables from the outset, not as subsequent combination rationales [195]. Using tumor lysates or multi-antigen loading is one approach to reduce single-epitope escape. However, breadth alone is insufficient if the local context remains suppressive. Here, a translationally coherent combination strategy involves pairing antigen-competent carriers with immune modulators that preserve effector function in tumors (for example, checkpoint blockade) and/or provide costimulatory support when priming is initiated. The key is to justify combinations mechanistically: the cell-based platform establishes antigen-specific T cells; the modulator prevents those T cells from being rapidly silenced or excluded in the tumor.

Safety, however, is where endogenous living adjuvants must clearly outperform their own complexity. For soluble agonists, toxicity is often dominated by systemic exposure and cytokine spillover [196]. Living systems can, in principle, narrow exposure by concentrating cues at cell-cell interfaces and restricting activity to defined compartments, but they can also introduce new hazards: unpredictable persistence, off-target accumulation, or context-driven immunosuppression (particularly for MSC-like carriers in chronic inflammation). Translational design should therefore incorporate self-limiting behavior as a deliberate feature, even when viability is retained. This can be achieved through controlling lifespan (short-lived carriers, irradiation when compatible with function), restricting activation to intended tissues, or using designs that allow clearance to end the stimulus before exhaustion or tolerance develops [197]. The guiding principle is not maximal persistence but matched persistence, as the stimulus should last long enough to establish expansion and memory, yet end before chronic signaling drives dysfunction.

In summary, translating endogenous living adjuvants into clinical practice requires a practical balance of manufacturability, persistence, and safety. To ensure manufacturability, the field must transition toward scalable, allogeneic cell sources and establish standardized potency assays to prevent batch-to-batch inconsistency. Persistence, meanwhile, should not be blindly maximized. Instead, the lifespan of the cellular carrier must be precisely matched to the time required for effective T-cell priming, as prolonged stimulation can lead to immune exhaustion or adaptive resistance. Ultimately, both production and persistence must prioritize safety. This requires designing platforms with controlled *in vivo* clearance, either by utilizing naturally short-lived cells or by engineering genetic shut-off switches, to ensure the immune response does not cause long-term systemic toxicity.

Looking forward, the most meaningful advances will be those that make endogenous living adjuvants easier to standardize while sharpening the reasons to use them. Three directions are likely to matter most. First, controllable shutoff: platforms that can be predictably cleared or that intrinsically time-limit their activation will expand the therapeutic window and simplify safety management. Second, APC-network targeting: designs that bias antigen handling toward cross-presenting dendritic cell subsets, rather than indiscriminate uptake by suppressive phagocytes, should improve the efficiency of priming per dose and reduce the need for high-intensity innate agonism. Third, modular manufacturing: a common chassis that can be loaded with patient-specific or indication-specific antigens without re-deriving the entire product each time would reduce batch variability and shorten manufacturing timelines. If these priorities are met, endogenous living adjuvants will justify their complexity not by being another way to add danger signals, but by making immune priming spatially directed, temporally shaped, and mechanistically verifiable, capabilities that conventional adjuvants struggle to provide in tumors and other anatomically-constrained immune-priming settings.

## 6 Conclusions

Endogenous living adjuvants broaden cancer vaccine design by coupling antigen availability with innate activation in a way that is constrained by trafficking routes and cellular processors. In conventional vaccines, adjuvant activity is largely mediated by soluble mediators and particulate uptake at the injection site, followed by antigen and danger signal drainage to the draining lymph node, where resident and recruited antigen-presenting cells (APCs) integrate these inputs. In living platforms, the “where” and “who” of immune sensing can be specified more directly because the carrier itself determines tissue distribution, the APC subset that acquires antigen, and the duration of antigen exposure. As a result, immune activation can be initiated in secondary lymphoid organs, the splenic clearance compartment, inflamed tissues, or within tumors, rather than being limited to the injection site-draining lymph node axis.

Across dendritic cell, B cell, erythrocyte, mesenchymal stromal cell, and tumor cell-based systems, a shared principle is that adjuvant effect is produced by controlling (1) the APC that processes antigen (*e.g.*, cDC1 *versus* macrophages), (2) the pattern-recognition receptor inputs and type I interferon-associated programs that accompany antigen uptake, and (3) the spatial microenvironment in which T cell priming occurs. These platforms also make common failure modes explicit. Once priming generates meaningful T cell pressure, tumors can escape *via* antigen loss and adaptive resistance programs. Therefore, durability depends on designing for antigen breadth and for maintaining effector function in the tumor microenvironment. Practically, this means pairing multi-antigen sources (*e.g.*, lysates or multi-epitope loading) with mechanistically matched immune modulators that prevent functional inactivation or exclusion of tumor-reactive T cells (for example, checkpoint blockade and costimulatory receptor agonism). If endogenous living adjuvants or carriers are engineered with controllable persistence and predictable clearance, they provide a tractable route to organ- and niche-selective immune modulation while limiting systemic cytokine exposure, offering immune outcomes that are difficult to reproduce with conventional adjuvant-antigen co-formulations.

## Ethics approval and consent to participate

This review article does not require any ethical approval or allied consent for publication.

## Funding source

This study was supported by the 10.13039/501100012166National Key R&D Program of China (2024YFA1212400), the 10.13039/501100001809National Natural Science Foundation of China (T2422023, 52173142), the “Pioneer” and “Leading Goose” R&D Program of Zhejiang (2024C03168), the Startup Package of 10.13039/501100004835Zhejiang University, the Zhejiang Provincial Health Science and Technology Plan (Clinical Research and Application Program, NO. 2022KY1301) and Shaoxing Municipal Health Science and Technology Plan (Open Laboratory Program, NO. 2022SY003).

## CRediT authorship contribution statement

**Jiaxin Yu:** Conceptualization, Investigation, Methodology, Validation, Visualization, Writing – original draft, Writing – review & editing. **Chaojie Zhu:** Conceptualization, Investigation, Methodology, Project administration, Supervision, Validation, Visualization, Writing – original draft, Writing – review & editing. **Hongjun Li:** Conceptualization, Funding acquisition, Project administration, Supervision, Validation, Writing – original draft, Writing – review & editing. **Feng Xu:** Conceptualization, Funding acquisition, Project administration, Supervision, Validation, Writing – original draft, Writing – review & editing.

## Declaration of competing interest

The authors declare that they have no known competing financial interests or personal relationships that could have appeared to influence the work reported in this paper.

## Data Availability

No data was used for the research described in the article.

## References

[1] Zaidi N., Jaffee E.M., Yarchoan M. (2025). Recent advances in therapeutic cancer vaccines. Nat. Rev. Cancer.

[2] Saxena M., Van Der Burg S.H., Melief C.J.M., Bhardwaj N. (2021). Therapeutic cancer vaccines. Nat. Rev. Cancer.

[3] Gulley J.L., Borre M., Vogelzang N.J., Ng S., Agarwal N., Parker C.C., Pook D.W., Rathenborg P., Flaig T.W., Carles J., Saad F., Shore N.D., Chen L., Heery C.R., Gerritsen W.R., Priou F., Langkilde N.C., Novikov A., Kantoff P.W. (2019). Phase III trial of PROSTVAC in asymptomatic or minimally symptomatic metastatic castration-resistant prostate cancer. J. Clin. Orthod..

[4] Lin M.J., Svensson-Arvelund J., Lubitz G.S., Marabelle A., Melero I., Brown B.D., Brody J.D. (2022). Cancer vaccines: the next immunotherapy frontier. Nat. Cancer.

[5] Kantoff P.W., Higano C.S., Shore N.D., Berger E.R., Small E.J., Penson D.F., Redfern C.H., Ferrari A.C., Dreicer R., Sims R.B., Xu Y., Frohlich M.W., Schellhammer P.F. (2010). Sipuleucel-T immunotherapy for castration-resistant prostate cancer. N. Engl. J. Med..

[6] Melief C.J.M., Van Hall T., Arens R., Ossendorp F., Van Der Burg S.H. (2015). Therapeutic cancer vaccines. J. Clin. Investig..

[7] Melief C.J.M., Van Der Burg S.H. (2008). Immunotherapy of established (pre)malignant disease by synthetic long peptide vaccines. Nat. Rev. Cancer.

[8] Fan T., Zhang M., Yang J., Zhu Z., Cao W., Dong C. (2023). Therapeutic cancer vaccines: advancements, challenges and prospects. Signal Transduct. Targeted Ther..

[9] Higgins J.P., Bernstein M.B., Hodge J.W. (2009). Enhancing immune responses to tumor-associated antigens. Cancer Biol. Ther..

[10] Coulie P.G., Van Den Eynde B.J., Van Der Bruggen P., Boon T. (2014). Tumour antigens recognized by T lymphocytes: at the core of cancer immunotherapy. Nat. Rev. Cancer.

[11] Sahin U., Derhovanessian E., Miller M., Kloke B.-P., Simon P., Löwer M., Bukur V., Tadmor A.D., Luxemburger U., Schrörs B., Omokoko T., Vormehr M., Albrecht C., Paruzynski A., Kuhn A.N., Buck J., Heesch S., Schreeb K.H., Müller F., Ortseifer I., Vogler I., Godehardt E., Attig S., Rae R., Breitkreuz A., Tolliver C., Suchan M., Martic G., Hohberger A., Sorn P., Diekmann J., Ciesla J., Waksmann O., Brück A.-K., Witt M., Zillgen M., Rothermel A., Kasemann B., Langer D., Bolte S., Diken M., Kreiter S., Nemecek R., Gebhardt C., Grabbe S., Höller C., Utikal J., Huber C., Loquai C., Türeci Ö. (2017). Personalized RNA mutanome vaccines mobilize poly-specific therapeutic immunity against cancer. Nature.

[12] Ott P.A., Hu Z., Keskin D.B., Shukla S.A., Sun J., Bozym D.J., Zhang W., Luoma A., Giobbie-Hurder A., Peter L., Chen C., Olive O., Carter T.A., Li S., Lieb D.J., Eisenhaure T., Gjini E., Stevens J., Lane W.J., Javeri I., Nellaiappan K., Salazar A.M., Daley H., Seaman M., Buchbinder E.I., Yoon C.H., Harden M., Lennon N., Gabriel S., Rodig S.J., Barouch D.H., Aster J.C., Getz G., Wucherpfennig K., Neuberg D., Ritz J., Lander E.S., Fritsch E.F., Hacohen N., Wu C.J. (2017). An immunogenic personal neoantigen vaccine for patients with melanoma. Nature.

[13] Cuzzubbo S., Mangsbo S., Nagarajan D., Habra K., Pockley A.G., McArdle S.E.B. (2021). Cancer vaccines: adjuvant potency, importance of age, lifestyle, and treatments. Front. Immunol..

[14] Bachmann M.F., Jennings G.T. (2010). Vaccine delivery: a matter of size, geometry, kinetics and molecular patterns. Nat. Rev. Immunol..

[15] Roth G.A., Picece V.C.T.M., Ou B.S., Luo W., Pulendran B., Appel E.A. (2021). Designing spatial and temporal control of vaccine responses. Nat. Rev. Mater..

[16] O'Hagan D.T., Valiante N.M. (2003). Recent advances in the discovery and delivery of vaccine adjuvants. Nat. Rev. Drug Discov..

[17] Coffman R.L., Sher A., Seder R.A. (2010). Vaccine adjuvants: putting innate immunity to work. Immunity.

[18] Zhao T., Cai Y., Jiang Y., He X., Wei Y., Yu Y., Tian X. (2023). Vaccine adjuvants: mechanisms and platforms. Signal Transduct. Targeted Ther..

[19] Lavelle E.C., McEntee C.P. (2024). Vaccine adjuvants: tailoring innate recognition to send the right message. Immunity.

[20] Mehta N.K., Moynihan K.D., Irvine D.J. (2015). Engineering new approaches to cancer vaccines. Cancer Immunol. Res..

[21] Najibi A.J., Mooney D.J. (2020). Cell and tissue engineering in lymph nodes for cancer immunotherapy. Adv. Drug Deliv. Rev..

[22] Lin Y.-J., Shih Y.-J., Chen C.-H., Fang C.-T. (2018). Aluminum salts as an adjuvant for pre-pandemic influenza vaccines: a meta-analysis. Sci. Rep..

[23] Coleman B.L., Sanderson R., Haag M.D.M., McGovern I. (2021). Effectiveness of the MF59‐adjuvanted trivalent or quadrivalent seasonal influenza vaccine among adults 65 years of age or older, a systematic review and meta‐analysis. Influenza Resp Virus.

[24] Paavonen J., Naud P., Salmerón J., Wheeler C.M., Chow S.N., Apter D., Kitchener H., Castellsague X., Teixeira J.C., Skinner S.R., Hedrick J., Jaisamrarn U., Limson G., Garland S., Szarewski A., Romanowski B., Aoki F.Y., Schwarz T.F., Poppe W.A., Bosch F.X., Jenkins D., Hardt K., Zahaf T., Descamps D., Struyf F., Lehtinen M., Dubin G. (2009). Efficacy of human papillomavirus (HPV)-16/18 AS04-adjuvanted vaccine against cervical infection and precancer caused by oncogenic HPV types (PATRICIA): final analysis of a double-blind, randomised study in young women. Lancet.

[25] Vansteenkiste J.F., Cho B.C., Vanakesa T., De Pas T., Zielinski M., Kim M.S., Jassem J., Yoshimura M., Dahabreh J., Nakayama H., Havel L., Kondo H., Mitsudomi T., Zarogoulidis K., Gladkov O.A., Udud K., Tada H., Hoffman H., Bugge A., Taylor P., Gonzalez E.E., Liao M.L., He J., Pujol J.-L., Louahed J., Debois M., Brichard V., Debruyne C., Therasse P., Altorki N. (2016). Efficacy of the MAGE-A3 cancer immunotherapeutic as adjuvant therapy in patients with resected MAGE-A3-positive non-small-cell lung cancer (MAGRIT): a randomised, double-blind, placebo-controlled, phase 3 trial. Lancet Oncol..

[26] Dreno B., Thompson J.F., Smithers B.M., Santinami M., Jouary T., Gutzmer R., Levchenko E., Rutkowski P., Grob J.-J., Korovin S., Drucis K., Grange F., Machet L., Hersey P., Krajsova I., Testori A., Conry R., Guillot B., Kruit W.H.J., Demidov L., Thompson J.A., Bondarenko I., Jaroszek J., Puig S., Cinat G., Hauschild A., Goeman J.J., Van Houwelingen H.C., Ulloa-Montoya F., Callegaro A., Dizier B., Spiessens B., Debois M., Brichard V.G., Louahed J., Therasse P., Debruyne C., Kirkwood J.M. (2018). MAGE-A3 immunotherapeutic as adjuvant therapy for patients with resected, MAGE-A3-positive, stage III melanoma (DERMA): a double-blind, randomised, placebo-controlled, phase 3 trial. Lancet Oncol..

[27] Harimoto T., Jung W.-H., Mooney D.J. (2025). Delivering living medicines with biomaterials. Nat. Rev. Mater..

[28] Shen X., Zhu C., Liu X., Zheng H., Wu Q., Xie J., Huang H., Liao Z., Shi J., Nan K., Wang J., Mao X., Gu Z., Li H. (2023). Engineered bacteria for augmented *In situ* tumor vaccination. Biomater. Sci..

[29] Shalhout S.Z., Miller D.M., Emerick K.S., Kaufman H.L. (2023). Therapy with oncolytic viruses: progress and challenges. Nat. Rev. Clin. Oncol..

[30] Gong N., Alameh M.-G., El-Mayta R., Xue L., Weissman D., Mitchell M.J. (2024). Enhancing in situ cancer vaccines using delivery technologies. Nat. Rev. Drug Discov..

[31] Helmink B.A., Reddy S.M., Gao J., Zhang S., Basar R., Thakur R., Yizhak K., Sade-Feldman M., Blando J., Han G., Gopalakrishnan V., Xi Y., Zhao H., Amaria R.N., Tawbi H.A., Cogdill A.P., Liu W., LeBleu V.S., Kugeratski F.G., Patel S., Davies M.A., Hwu P., Lee J.E., Gershenwald J.E., Lucci A., Arora R., Woodman S., Keung E.Z., Gaudreau P.-O., Reuben A., Spencer C.N., Burton E.M., Haydu L.E., Lazar A.J., Zapassodi R., Hudgens C.W., Ledesma D.A., Ong S., Bailey M., Warren S., Rao D., Krijgsman O., Rozeman E.A., Peeper D., Blank C.U., Schumacher T.N., Butterfield L.H., Zelazowska M.A., McBride K.M., Kalluri R., Allison J., Petitprez F., Fridman W.H., Sautès-Fridman C., Hacohen N., Rezvani K., Sharma P., Tetzlaff M.T., Wang L., Wargo J.A. (2020). B cells and tertiary lymphoid structures promote immunotherapy response. Nature.

[32] Zhang L., Sun X., Jia Y., Liu X., Dong M., Xu Z.P., Liu R. (2020). Nanovaccine's rapid induction of anti-tumor immunity significantly improves malignant cancer immunotherapy. Nano Today.

[33] Zhang L.-X., Hu J., Jia Y.-B., Liu R.-T., Cai T., Xu Z.P. (2021). Two-dimensional layered double hydroxide nanoadjuvant: recent progress and future direction. Nanoscale.

[34] Zhang L., Xie X., Liu D., Xu Z.P., Liu R. (2018). Efficient co-delivery of neo-epitopes using dispersion-stable layered double hydroxide nanoparticles for enhanced melanoma immunotherapy. Biomaterials.

[35] Detienne S., Welsby I., Collignon C., Wouters S., Coccia M., Delhaye S., Van Maele L., Thomas S., Swertvaegher M., Detavernier A., Elouahabi A., Goriely S., Didierlaurent A.M. (2016). Central role of CD169+ lymph node resident macrophages in the adjuvanticity of the QS-21 component of AS01. Sci. Rep..

[36] Grabowska J., Lopez-Venegas M.A., Affandi A.J., Den Haan J.M.M. (2018). CD169+ macrophages capture and dendritic cells instruct: the interplay of the gatekeeper and the general of the immune system. Front. Immunol..

[37] Martín-Fontecha A., Sebastiani S., Höpken U.E., Uguccioni M., Lipp M., Lanzavecchia A., Sallusto F. (2003). Regulation of dendritic cell migration to the draining lymph node. J. Exp. Med..

[38] Yousefpour P., Ni K., Irvine D.J. (2023). Targeted modulation of immune cells and tissues using engineered biomaterials. Nat. Rev. Bioeng..

[39] Bozzacco L., Trumpfheller C., Siegal F.P., Mehandru S., Markowitz M., Carrington M., Nussenzweig M.C., Piperno A.G., Steinman R.M. (2007). DEC-205 receptor on dendritic cells mediates presentation of HIV gag protein to CD8^+^ T cells in a spectrum of human MHC I haplotypes. Proc. Natl. Acad. Sci. USA.

[40] Lee D., Huntoon K., Lux J., Kim B.Y.S., Jiang W. (2023). Engineering nanomaterial physical characteristics for cancer immunotherapy. Nat. Rev. Bioeng..

[41] León B., Lund F.E. (2019). Compartmentalization of dendritic cell and t‐cell interactions in the lymph node: anatomy of t‐cell fate decisions. Immunol. Rev..

[42] Lewis S.M., Williams A., Eisenbarth S.C. (2019). Structure and function of the immune system in the spleen. Sci. Immunol..

[43] Calabro S., Liu D., Gallman A., Nascimento M.S.L., Yu Z., Zhang T., Chen P., Zhang B., Xu L., Gowthaman U., Krishnaswamy J.K., Haberman A.M., Williams A., Eisenbarth S.C. (2016). Differential intrasplenic migration of dendritic cell subsets tailors adaptive immunity. Cell Rep..

[44] Kranz L.M., Diken M., Haas H., Kreiter S., Loquai C., Reuter K.C., Meng M., Fritz D., Vascotto F., Hefesha H., Grunwitz C., Vormehr M., Hüsemann Y., Selmi A., Kuhn A.N., Buck J., Derhovanessian E., Rae R., Attig S., Diekmann J., Jabulowsky R.A., Heesch S., Hassel J., Langguth P., Grabbe S., Huber C., Türeci Ö., Sahin U. (2016). Systemic RNA delivery to dendritic cells exploits antiviral defence for cancer immunotherapy. Nature.

[45] Toussi D., Massari P. (2014). Immune adjuvant effect of molecularly-defined toll-like receptor ligands. Vaccines.

[46] Sánchez-Paulete A.R., Teijeira A., Cueto F.J., Garasa S., Pérez-Gracia J.L., Sánchez-Arráez A., Sancho D., Melero I. (2017). Antigen cross-presentation and T-cell cross-priming in cancer immunology and immunotherapy. Ann. Oncol..

[47] Khleif S.N., Gupta S. (2025). Cancer vaccines as enablers of immunotherapy. Nat. Immunol..

[48] Luri-Rey C., Teijeira Á., Wculek S.K., De Andrea C., Herrero C., Lopez-Janeiro A., Rodríguez-Ruiz M.E., Heras I., Aggelakopoulou M., Berraondo P., Sancho D., Melero I. (2025). Cross-priming in cancer immunology and immunotherapy. Nat. Rev. Cancer.

[49] Rojas L.A., Sethna Z., Soares K.C., Olcese C., Pang N., Patterson E., Lihm J., Ceglia N., Guasp P., Chu A., Yu R., Chandra A.K., Waters T., Ruan J., Amisaki M., Zebboudj A., Odgerel Z., Payne G., Derhovanessian E., Müller F., Rhee I., Yadav M., Dobrin A., Sadelain M., Łuksza M., Cohen N., Tang L., Basturk O., Gönen M., Katz S., Do R.K., Epstein A.S., Momtaz P., Park W., Sugarman R., Varghese A.M., Won E., Desai A., Wei A.C., D'Angelica M.I., Kingham T.P., Mellman I., Merghoub T., Wolchok J.D., Sahin U., Türeci Ö., Greenbaum B.D., Jarnagin W.R., Drebin J., O'Reilly E.M., Balachandran V.P. (2023). Personalized RNA neoantigen vaccines stimulate T cells in pancreatic cancer. Nature.

[50] Alloatti A., Kotsias F., Magalhaes J.G., Amigorena S. (2016). Dendritic cell maturation and cross‐presentation: timing matters. Immunol. Rev..

[51] Lurje I., Werner W., Mohr R., Roderburg C., Tacke F., Hammerich L. (2021). In situ vaccination as a strategy to modulate the immune microenvironment of hepatocellular carcinoma. Front. Immunol..

[52] Hammerich L., Binder A., Brody J.D. (2015). *In situ* vaccination: cancer immunotherapy both personalized *and* off‐the‐shelf. Mol. Oncol..

[53] Zhang L., Zhao J., Hu X., Wang C., Jia Y., Zhu C., Xie S., Lee J., Li F., Ling D. (2022). A peritumorally injected immunomodulating adjuvant elicits robust and safe metalloimmunotherapy against solid tumors. Adv. Mater..

[54] Zhu C., Liu C., Wu Q., Sheng T., Zhou R., Ren E., Zhang R., Zhao Z., Shi J., Shen X., Sun Z., Mao Z., He K., Zhang L., Ding Y., Gu Z., Wang W., Li H. (2024). Remolding the tumor microenvironment by bacteria augments adoptive T cell therapy in advanced-stage solid tumors. Signal Transduct. Targeted Ther..

[55] Arina A., Gutiontov S.I., Weichselbaum R.R. (2020). Radiotherapy and immunotherapy for cancer: from “Systemic” to “Multisite,”. Clin. Cancer Res..

[56] Hernandez C., Huebener P., Schwabe R.F. (2016). Damage-associated molecular patterns in cancer: a double-edged sword. Oncogene.

[57] Zhang L., Jia Y., Yang J., Zhang L., Hou S., Niu X., Zhu J., Huang Y., Sun X., Xu Z.P., Liu R. (2022). Efficient immunotherapy of drug-free layered double hydroxide nanoparticles via neutralizing excess acid and blocking tumor cell autophagy. ACS Nano.

[58] Andtbacka R.H.I., Kaufman H.L., Collichio F., Amatruda T., Senzer N., Chesney J., Delman K.A., Spitler L.E., Puzanov I., Agarwala S.S., Milhem M., Cranmer L., Curti B., Lewis K., Ross M., Guthrie T., Linette G.P., Daniels G.A., Harrington K., Middleton M.R., Miller W.H., Zager J.S., Ye Y., Yao B., Li A., Doleman S., VanderWalde A., Gansert J., Coffin R.S. (2015). Talimogene laherparepvec improves durable response rate in patients with advanced melanoma. J. Clin. Orthod..

[59] Gamrekelashvili J., Greten T.F., Korangy F. (2015). Immunogenicity of necrotic cell death. Cell. Mol. Life Sci..

[60] Paardekooper L.M., Vos W., Van Den Bogaart G. (2019). Oxygen in the tumor microenvironment: effects on dendritic cell function. Oncotarget.

[61] Gupta Y.H., Khanom A., Acton S.E. (2022). Control of dendritic cell function within the tumour microenvironment. Front. Immunol..

[62] Du Bois H., Heim T.A., Lund A.W. (2021). Tumor-draining lymph nodes: at the crossroads of metastasis and immunity. Sci. Immunol..

[63] Pittet M.J., Di Pilato M., Garris C., Mempel T.R. (2023). Dendritic cells as shepherds of T cell immunity in cancer. Immunity.

[64] Marabelle A., Tselikas L., De Baere T., Houot R. (2017). Intratumoral immunotherapy: using the tumor as the remedy. Ann. Oncol..

[65] Reed S.G., Orr M.T., Fox C.B. (2013). Key roles of adjuvants in modern vaccines. Nat. Med..

[66] Pulendran B., Arunachalam P.S., O'Hagan D.T. (2021). Emerging concepts in the science of vaccine adjuvants. Nat. Rev. Drug Discov..

[67] Li Z., Wang Y., Gu Z., Hu Q. (2024). Engineering cells for therapy and diagnosis. Nat. Rev. Bioeng..

[68] Banchereau J., Steinman R.M. (1998). Dendritic cells and the control of immunity. Nature.

[69] Tan M.C.A.A., Mommaas A.M., Drijfhout J.W., Jordens R., Onderwater J.J.M., Verwoerd D., Mulder A.A., Van Der Heiden A.N., Scheidegger D., Oomen L.C.J.M., Ottenhoff T.H.M., Tulp A., Neefjes J.J., Koning F. (1997). Mannose receptor‐mediated uptake of antigens strongly enhances HLA class II‐restricted antigen presentation by cultured dendritic cells. Eur. J. Immunol..

[70] Platt C.D., Ma J.K., Chalouni C., Ebersold M., Bou-Reslan H., Carano R.A.D., Mellman I., Delamarre L. (2010). Mature dendritic cells use endocytic receptors to capture and present antigens. Proc. Natl. Acad. Sci. USA.

[71] Kim M.K., Kim J. (2019). Properties of immature and mature dendritic cells: phenotype, morphology, phagocytosis, and migration. RSC Adv..

[72] Garcia J.A. (2011). Sipuleucel-T in patients with metastatic castration-resistant prostate cancer: an insight for oncologists. Ther. Adv. Med. Oncol..

[73] Cheever M.A., Higano C.S. (2011). PROVENGE (Sipuleucel-T) in prostate cancer: the first FDA-approved therapeutic cancer vaccine. Clin. Cancer Res..

[74] Sheikh N.A., Petrylak D., Kantoff P.W., Dela Rosa C., Stewart F.P., Kuan L.-Y., Whitmore J.B., Trager J.B., Poehlein C.H., Frohlich M.W., Urdal D.L. (2013). Sipuleucel-T immune parameters correlate with survival: an analysis of the randomized phase 3 clinical trials in men with castration-resistant prostate cancer. Cancer Immunol. Immunother..

[75] Shen X., Zhu C., Wu Q., Shi J., Wu W., Zhao X., Sun J., Li H., Gu Z. (2022). Nanomodulators targeting tumor-resident immunosuppressive cells: mechanisms and recent updates. Nano Today.

[76] Palucka K., Banchereau J. (2013). Dendritic-cell-based therapeutic cancer vaccines. Immunity.

[77] Sabado R.L., Balan S., Bhardwaj N. (2017). Dendritic cell-based immunotherapy. Cell Res..

[78] Bol K.F., Schreibelt G., Rabold K., Wculek S.K., Schwarze J.K., Dzionek A., Teijeira A., Kandalaft L.E., Romero P., Coukos G., Neyns B., Sancho D., Melero I., De Vries I.J.M. (2019). The clinical application of cancer immunotherapy based on naturally circulating dendritic cells. J. Immunother. Cancer.

[79] Ridolfi R., Riccobon A., Galassi R., Giorgetti G., Petrini M., Fiammenghi L., Stefanelli M., Ridolfi L., Moretti A., Migliori G., Fiorentini G. (2004). Evaluation of in vivo labelled dendritic cell migration in cancer patients. J. Transl. Med..

[80] Jin H., Qian Y., Dai Y., Qiao S., Huang C., Lu L., Luo Q., Chen J., Zhang Z. (2016). Magnetic enrichment of dendritic cell vaccine in lymph node with fluorescent-magnetic nanoparticles enhanced cancer immunotherapy. Theranostics.

[81] Yu L., Feng R., Zhu L., Hao Q., Chu J., Gu Y., Luo Y., Zhang Z., Chen G., Chen H. (2020). Promoting the activation of T cells with glycopolymer-modified dendritic cells by enhancing cell interactions. Sci. Adv..

[82] Hu S., Yi W., Zhao Z., Qian X., Jiang L., Cao Y., Yan D., Teng L., Li Y. (2025). A lymph node-targeted cell-nanoadjuvant conjugate enhances dendritic cell-T cell crosstalk for cancer immunotherapy. Acta Pharm. Sin. B.

[83] Yi W., Qian X., Yan D., Yan W., Hu S., Li Y., Wang D. (2025). A dendritic cell‐nanogel conjugate for tumor‐draining lymph node‐specific PD‐L1 blockade. Adv. Mater..

[84] Han J., Bhatta R., Liu Y., Bo Y., Elosegui-Artola A., Wang H. (2023). Metabolic glycan labeling immobilizes dendritic cell membrane and enhances antitumor efficacy of dendritic cell vaccine. Nat. Commun..

[85] Van Nuffel A.M., Benteyn D., Wilgenhof S., Pierret L., Corthals J., Heirman C., Van Der Bruggen P., Coulie P.G., Neyns B., Thielemans K., Bonehill A. (2012). Dendritic cells loaded with mRNA encoding full-length tumor antigens prime CD4+ and CD8+ T cells in melanoma patients. Mol. Ther..

[86] Wilgenhof S., Van Nuffel A.M.T., Benteyn D., Corthals J., Aerts C., Heirman C., Van Riet I., Bonehill A., Thielemans K., Neyns B. (2013). A phase IB study on intravenous synthetic mRNA electroporated dendritic cell immunotherapy in pretreated advanced melanoma patients. Ann. Oncol..

[87] Aarntzen E.H.J.G., Schreibelt G., Bol K., Lesterhuis W.J., Croockewit A.J., De Wilt J.H.W., Van Rossum M.M., Blokx W.A.M., Jacobs J.F.M., Duiveman-de Boer T., Schuurhuis D.H., Mus R., Thielemans K., De Vries I.J.M., Figdor C.G., Punt C.J.A., Adema G.J. (2012). Vaccination with mRNA-Electroporated dendritic cells induces robust tumor antigen-specific CD4+ and CD8+ T cells responses in stage III and IV melanoma patients. Clin. Cancer Res..

[88] Ménager J., Ebstein F., Oger R., Hulin P., Nedellec S., Duverger E., Lehmann A., Kloetzel P.-M., Jotereau F., Guilloux Y. (2014). Cross-presentation of synthetic long peptides by human dendritic cells: a process dependent on ERAD component p97/VCP but not sec61 and/or Derlin-1. PLoS One.

[89] Cafri G., Sharbi-Yunger A., Tzehoval E., Alteber Z., Gross T., Vadai E., Margalit A., Gross G., Eisenbach L. (2015). mRNA-transfected dendritic cells expressing polypeptides that link MHC-I presentation to constitutive TLR4 activation confer tumor immunity. Mol. Ther..

[90] Ponsaerts P., Van Tendeloo V.F.I., Berneman Z.N. (2003). Cancer immunotherapy using RNA-loaded dendritic cells. Clin. Exp. Immunol..

[91] Boudreau J.E., Bonehill A., Thielemans K., Wan Y. (2011). Engineering dendritic cells to enhance cancer immunotherapy. Mol. Ther..

[92] Barbier A.J., Jiang A.Y., Zhang P., Wooster R., Anderson D.G. (2022). The clinical progress of mRNA vaccines and immunotherapies. Nat. Biotechnol..

[93] Kon E., Ad-El N., Hazan-Halevy I., Stotsky-Oterin L., Peer D. (2023). Targeting cancer with mRNA–lipid nanoparticles: key considerations and future prospects. Nat. Rev. Clin. Oncol..

[94] Zhang H., Meng C., Yi X., Han J., Wang J., Liu F., Ling Q., Li H., Gu Z. (2024). Fluorinated lipid nanoparticles for enhancing mRNA delivery efficiency. ACS Nano.

[95] Meng C., Zhang H., Yi X., Kong G., Zhang X., Wang B., Xu Y., Qi H., Wu Q., Zhang K., Cao J., Lin X., Feng H., Chen J., Zheng S., Gu Z., Li H., Ling Q. (2025). Reversal of tumour immune evasion via enhanced MHC-Class-I antigen presentation by a dual-functional RNA regulated system. Mol. Cancer.

[96] Hobo W., Novobrantseva T.I., Fredrix H., Wong J., Milstein S., Epstein-Barash H., Liu J., Schaap N., Van Der Voort R., Dolstra H. (2013). Improving dendritic cell vaccine immunogenicity by silencing PD-1 ligands using siRNA-lipid nanoparticles combined with antigen mRNA electroporation. Cancer Immunol. Immunother..

[97] Yang D., Zhan J., Miao L., He C., Xu H. (2025). Lipid nanoparticle-mediated mRNA/siRNA dual-bioengineered dendritic cell vaccines combined of PD-1/PD-L1 blockade for boosting tumor immunotherapy. Mater. Today Bio.

[98] Chao C.-J., Zhang E., Trinh D.N., Udofa E., Lin H., Silvers C., Huo J., He S., Zheng J., Cai X., Bao Q., Zhang L., Phan P., Elgendy S.M., Shi X., Burdette J.E., Lee S.S.-Y., Gao Y., Zhang P., Zhao Z. (2025). Integrating antigen capturing nanoparticles and type 1 conventional dendritic cell therapy for in situ cancer immunization. Nat. Commun..

[99] Zheng J., Li X., He A., Zhang Y., Yang Y., Dang M., Li Q., Mou Y., Dong H. (2025). In situ antigen-capture strategies for enhancing dendritic cell-mediated anti-tumor immunity. J. Contr. Release.

[100] McHeyzer-Williams L.J., Pelletier N., Mark L., Fazilleau N., McHeyzer-Williams M.G. (2009). Follicular helper T cells as cognate regulators of B cell immunity. Curr. Opin. Immunol..

[101] King C. (2011). A fine romance: t follicular helper cells and B cells. Immunity.

[102] Rastogi I., Jeon D., Moseman J.E., Muralidhar A., Potluri H.K., McNeel D.G. (2022). Role of B cells as antigen presenting cells. Front. Immunol..

[103] Fahlquist-Hagert C., Wittenborn T.R., Terczyńska-Dyla E., Kastberg K.S., Yang E., Rallistan A.N., Markett Q.R., Winther G., Fonager S., Voss L.F., Pedersen M.K., Van Campen N., Ferapontov A., Jensen L., Huang J., Nieland J.D., Van Der Poel C.E., Palmfeldt J., Carroll M.C., Utz P.J., Luo Y., Lin L., Degn S.E. (2023). Antigen presentation by B cells enables epitope spreading across an MHC barrier. Nat. Commun..

[104] Dunkelberger J.R., Song W.-C. (2010). Complement and its role in innate and adaptive immune responses. Cell Res..

[105] Alsén S., Cervin J., Deng Y., Szeponik L., Wenzel U.A., Karlsson J., Cucak H., Livingston M., Bryder D., Lu Q., Johansson-Lindbom B., Yrlid U. (2022). Antigen-presenting B cells program the efferent lymph T helper cell response. Front. Immunol..

[106] Lemoine S., Morva A., Youinou P., Jamin C. (2011). Human T cells induce their own regulation through activation of B cells. J. Autoimmun..

[107] Gadjalova I., Heinze J.M., Goess M.C., Hofmann J., Buck A., Weber M.-C., Blissenbach B., Kampick M., Krut O., Steiger K., Janssen K.-P., Neumann P.-A., Ruland J., Keppler S.J. (2024). B cell-mediated CD4 T-cell costimulation via CD86 exacerbates pro-inflammatory cytokine production during autoimmune intestinal inflammation. Mucosal Immunol..

[108] Okada T., Ngo V.N., Ekland E.H., Förster R., Lipp M., Littman D.R., Cyster J.G. (2002). Chemokine requirements for B cell entry to lymph nodes and peyer's patches. J. Exp. Med..

[109] Ohl L., Henning G., Krautwald S., Lipp M., Hardtke S., Bernhardt G., Pabst O., Förster R. (2003). Cooperating mechanisms of CXCR5 and CCR7 in development and organization of secondary lymphoid organs. J. Exp. Med..

[110] Lo C.G., Lu T.T., Cyster J.G. (2003). Integrin-dependence of lymphocyte entry into the splenic white pulp. J. Exp. Med..

[111] Grayson M.H., Chaplin D.D. (2003). Localization of T and B lymphocytes to the white pulp of the spleen is independent of L-, E-, and P-Selectin. Sci. World J..

[112] Corinti S., Medaglini D., Prezzi C., Cavani A., Pozzi G., Girolomoni G. (2000). Human dendritic cells are superior to B cells at presenting a major histocompatibility complex class II-Restricted heterologous antigen expressed on recombinant *Streptococcus gordonii*. Infect. Immun..

[113] Kawaguchi Y., Shimizu T., Takata H., Ando H., Ishida T. (2025). An in vitro nanocarrier-based B cell antigen loading system; tumor growth suppression via transfusion of the antigen-loaded B cells in vivo. Int. J. Pharm..

[114] Szeto G.L., Van Egeren D., Worku H., Sharei A., Alejandro B., Park C., Frew K., Brefo M., Mao S., Heimann M., Langer R., Jensen K., Irvine D.J. (2015). Microfluidic squeezing for intracellular antigen loading in polyclonal B-cells as cellular vaccines. Sci. Rep..

[115] Zhang L., Bridle B.W., Chen L., Pol J., Spaner D., Boudreau J.E., Rosen A., Bassett J.D., Lichty B.D., Bramson J.L., Wan Y. (2013). Delivery of viral-vectored vaccines by B cells represents a novel strategy to accelerate CD8+ T-cell recall responses. Blood.

[116] Embgenbroich M., Burgdorf S. (2018). Current concepts of antigen cross-presentation. Front. Immunol..

[117] Li Q., Lao X., Pan Q., Ning N., Yet J., Xu Y., Li S., Chang A.E. (2011). Adoptive transfer of tumor reactive B cells confers host T-Cell immunity and tumor regression. Clin. Cancer Res..

[118] Platzer B., Stout M., Fiebiger E. (2014). Antigen cross-presentation of immune complexes. Front. Immunol..

[119] Nguyen P.H.D., Jayasinghe M.K., Le A.H., Peng B., Le M.T.N. (2023). Advances in drug delivery systems based on red blood cells and their membrane-derived nanoparticles. ACS Nano.

[120] Alam N., Farrell B., Jamwal A., Higgins M.K. (2026). Erythrocyte invasion in malaria: from molecular mechanisms to rational vaccines. Nat. Rev. Microbiol..

[121] Anselmo A.C., Gupta V., Zern B.J., Pan D., Zakrewsky M., Muzykantov V., Mitragotri S. (2013). Delivering nanoparticles to lungs while avoiding liver and spleen through adsorption on red blood cells. ACS Nano.

[122] Brenner J.S., Pan D.C., Myerson J.W., Marcos-Contreras O.A., Villa C.H., Patel P., Hekierski H., Chatterjee S., Tao J.-Q., Parhiz H., Bhamidipati K., Uhler T.G., Hood E.D., Kiseleva R.Yu, Shuvaev V.S., Shuvaeva T., Khoshnejad M., Johnston I., Gregory J.V., Lahann J., Wang T., Cantu E., Armstead W.M., Mitragotri S., Muzykantov V. (2018). Red blood cell-hitchhiking boosts delivery of nanocarriers to chosen organs by orders of magnitude. Nat. Commun..

[123] Schmidt C.K., Medina-Sánchez M., Edmondson R.J., Schmidt O.G. (2020). Engineering microrobots for targeted cancer therapies from a medical perspective. Nat. Commun..

[124] Kontos S., Kourtis I.C., Dane K.Y., Hubbell J.A. (2013). Engineering antigens for in situ erythrocyte binding induces T-cell deletion. Proc. Natl. Acad. Sci. USA.

[125] Wu M., Luo Z., Cai Z., Mao Q., Li Z., Li H., Zhang C., Zhang Y., Zhong A., Wu L., Liu X. (2023). Spleen‐targeted neoantigen DNA vaccine for personalized immunotherapy of hepatocellular carcinoma. EMBO Mol. Med..

[126] Liu Y., Nie X., Yao X., Shou H., Yuan Y., Ge Y., Tong X., Lee H.-Y., Gao X. (2024). Developing an erythrocyte‒MHC-I conjugate for cancer treatment. Cell Discov..

[127] Ukidve A., Zhao Z., Fehnel A., Krishnan V., Pan D.C., Gao Y., Mandal A., Muzykantov V., Mitragotri S. (2020). Erythrocyte-driven immunization via biomimicry of their natural antigen-presenting function. Proc. Natl. Acad. Sci. USA.

[128] Zhao Z., Ukidve A., Krishnan V., Fehnel A., Pan D.C., Gao Y., Kim J., Evans M.A., Mandal A., Guo J., Muzykantov V.R., Mitragotri S. (2020). Systemic tumour suppression via the preferential accumulation of erythrocyte-anchored chemokine-encapsulating nanoparticles in lung metastases. Nat. Biomed. Eng..

[129] Salem H.K., Thiemermann C. (2010). Mesenchymal stromal cells: current understanding and clinical status. Stem Cell..

[130] Han X., Liao R., Li X., Zhang C., Huo S., Qin L., Xiong Y., He T., Xiao G., Zhang T. (2025). Mesenchymal stem cells in treating human diseases: molecular mechanisms and clinical studies. Signal Transduct. Targeted Ther..

[131] Le Blanc K., Rasmusson I., Sundberg B., Götherström C., Hassan M., Uzunel M., Ringdén O. (2004). Treatment of severe acute graft-versus-host disease with third party haploidentical mesenchymal stem cells. Lancet.

[132] Wang M., Yuan Q., Xie L. (2018). Mesenchymal stem cell-based immunomodulation: properties and clinical application. Stem Cell. Int..

[133] Sirpilla O., Sakemura R.L., Hefazi M., Huynh T.N., Can I., Girsch J.H., Tapper E.E., Cox M.J., Schick K.J., Manriquez-Roman C., Yun K., Stewart C.M., Ogbodo E.J., Kimball B.L., Mai L.K., Gutierrez-Ruiz O.L., Rodriguez M.L., Gluscevic M., Larson D.P., Abel A.M., Wierson W.A., Olivier G., Siegler E.L., Kenderian S.S. (2024). Mesenchymal stromal cells with chimaeric antigen receptors for enhanced immunosuppression. Nat. Biomed. Eng..

[134] Menon L.G., Shi V.J., Carroll R.S. (2009). Mesenchymal stromal cells as a drug delivery system. StemBook.

[135] Yin P., Gui L., Wang C., Yan J., Liu M., Ji L., Wang Y., Ma B., Gao W.-Q. (2020). Targeted delivery of CXCL9 and OX40L by mesenchymal stem cells elicits potent antitumor immunity. Mol. Ther..

[136] Abusarah J., Khodayarian F., El-Hachem N., Salame N., Olivier M., Balood M., Roversi K., Talbot S., Bikorimana J.-P., Chen J., Jolicoeur M., Trudeau L.-E., Kamyabiazar S., Annabi B., Robert F., Pelletier J., El-Kadiry A.-E.-H., Shammaa R., Rafei M. (2021). Engineering immunoproteasome-expressing mesenchymal stromal cells: a potent cellular vaccine for lymphoma and melanoma in mice. Cell Rep. Med..

[137] Cantero M.J., Bueloni B., Gonzalez Llamazares L., Fiore E., Lameroli L., Atorrasagasti C., Mazzolini G., Malvicini M., Bayo J., García M.G. (2024). Modified mesenchymal stromal cells by in vitro transcribed mRNA: a therapeutic strategy for hepatocellular carcinoma. Stem Cell Res. Ther..

[138] Chiang C.L.-L., Kandalaft L.E., Coukos G. (2011). Adjuvants for enhancing the immunogenicity of whole tumor cell vaccines. Int. Rev. Immunol..

[139] Diao L., Liu M. (2023). Rethinking antigen source: cancer vaccines based on whole tumor cell/Tissue lysate or whole tumor cell. Adv. Sci..

[140] Dranoff G., Jaffee E., Lazenby A., Golumbek P., Levitsky H., Brose K., Jackson V., Hamada H., Pardoll D., Mulligan R.C. (1993). Vaccination with irradiated tumor cells engineered to secrete murine granulocyte-macrophage colony-stimulating factor stimulates potent, specific, and long-lasting anti-tumor immunity. Proc. Natl. Acad. Sci. USA.

[141] Nemunaitis J., Dillman R.O., Schwarzenberger P.O., Senzer N., Cunningham C., Cutler J., Tong A., Kumar P., Pappen B., Hamilton C., DeVol E., Maples P.B., Liu L., Chamberlin T., Shawler D.L., Fakhrai H. (2006). Phase II study of Belagenpumatucel-L, a transforming growth factor Beta-2 antisense gene-modified allogeneic tumor cell vaccine in Non–small-cell lung cancer. J. Clin. Orthod..

[142] Giaccone G., Bazhenova L.A., Nemunaitis J., Tan M., Juhász E., Ramlau R., Van Den Heuvel M.M., Lal R., Kloecker G.H., Eaton K.D., Chu Q., Dunlop D.J., Jain M., Garon E.B., Davis C.S., Carrier E., Moses S.C., Shawler D.L., Fakhrai H. (2015). A phase III study of belagenpumatucel-L, an allogeneic tumour cell vaccine, as maintenance therapy for non-small cell lung cancer. Eur. J. Cancer.

[143] Ci T., Li H., Chen G., Wang Z., Wang J., Abdou P., Tu Y., Dotti G., Gu Z. (2020). Cryo-shocked cancer cells for targeted drug delivery and vaccination. Sci. Adv..

[144] Parkins K.M., Dubois V.P., Kelly J.J., Chen Y., Knier N.N., Foster P.J., Ronald J.A. (2020). Engineering circulating tumor cells as novel cancer theranostics. Theranostics.

[145] Liu F., Xin M., Feng H., Zhang W., Liao Z., Sheng T., Wen P., Wu Q., Liang T., Shi J., Zhou R., He K., Gu Z., Li H. (2024). Cryo-shocked tumor cells deliver CRISPR-Cas9 for lung cancer regression by synthetic lethality. Sci. Adv..

[146] Wu Q., Huang H., Sun M., Zhang R., Wang J., Zheng H., Zhu C., Yang S., Shen X., Shi J., Liu F., Wu W., Sun J., Liu F., Li H., Gu Z. (2023). Inhibition of tumor metastasis by liquid‐nitrogen‐shocked tumor cells with oncolytic viruses infection. Adv. Mater..

[147] Palucka K., Banchereau J. (2012). Cancer immunotherapy via dendritic cells. Nat. Rev. Cancer.

[148] Böttcher J.P., Reis E Sousa C. (2018). The role of type 1 conventional dendritic cells in cancer immunity. Trends Cancer.

[149] Kerfoot S.M., Yaari G., Patel J.R., Johnson K.L., Gonzalez D.G., Kleinstein S.H., Haberman A.M. (2011). Germinal center B cell and T follicular helper cell development initiates in the interfollicular zone. Immunity.

[150] Villa C.H., Anselmo A.C., Mitragotri S., Muzykantov V. (2016). Red blood cells: supercarriers for drugs, biologicals, and nanoparticles and inspiration for advanced delivery systems. Adv. Drug Deliv. Rev..

[151] Shi Y., Zhang J., Li Y., Feng C., Shao C., Shi Y., Fang J. (2025). Engineered mesenchymal stem/stromal cells against cancer. Cell Death Dis..

[152] Tseng S.-Y., Waite J.C., Liu M., Vardhana S., Dustin M.L. (2008). T cell-dendritic cell immunological synapses contain TCR-dependent CD28-CD80 clusters that recruit protein kinase Cθ. J. Immunol..

[153] Engel A.L., Holt G.E., Lu H. (2011). The pharmacokinetics of toll-like receptor agonists and the impact on the immune system. Expet Rev. Clin. Pharmacol..

[154] Brzostek J., Gascoigne N.R.J., Rybakin V. (2016). Cell type-specific regulation of immunological synapse dynamics by B7 ligand recognition. Front. Immunol..

[155] Lee K.-W., Yam J.W.P., Mao X. (2023). Dendritic cell vaccines: a shift from conventional approach to new generations. Cells.

[156] Iida Y., Yoshikawa R., Murata A., Kotani H., Kazuki Y., Oshimura M., Matsuzaki Y., Harada M. (2020). Local injection of CCL19-expressing mesenchymal stem cells augments the therapeutic efficacy of anti-PD-L1 antibody by promoting infiltration of immune cells. J. Immunother. Cancer.

[157] Irvine D.J., Aung A., Silva M. (2020). Controlling timing and location in vaccines. Adv. Drug Deliv. Rev..

[158] Langenkamp A., Messi M., Lanzavecchia A., Sallusto F. (2000). Kinetics of dendritic cell activation: impact on priming of TH1, TH2 and nonpolarized T cells. Nat. Immunol..

[159] Doyen V., Rubio M., Braun D., Nakajima T., Abe J., Saito H., Delespesse G., Sarfati M. (2003). Thrombospondin 1 is an autocrine negative regulator of human dendritic cell activation. J. Exp. Med..

[160] Wherry E.J. (2011). T cell exhaustion. Nat. Immunol..

[161] Floyd T.L., Koehn B.H., Kitchens W.H., Robertson J.M., Cheeseman J.A., Stempora L., Larsen C.P., Ford M.L. (2011). Limiting the amount and duration of antigen exposure during priming increases memory T cell requirement for costimulation during recall. J. Immunol..

[162] Cockburn I.A., Chen Y.-C., Overstreet M.G., Lees J.R., Van Rooijen N., Farber D.L., Zavala F. (2010). Prolonged antigen presentation is required for optimal CD8+ T cell responses against malaria liver stage parasites. PLoS Pathog..

[163] Yewdall A.W., Drutman S.B., Jinwala F., Bahjat K.S., Bhardwaj N. (2010). CD8+ T cell priming by dendritic cell vaccines requires antigen transfer to endogenous antigen presenting cells. PLoS One.

[164] Camporeale A., Boni A., Iezzi G., Degl'Innocenti E., Grioni M., Mondino A., Bellone M. (2003). Critical impact of the kinetics of dendritic cells activation on the in vivo induction of tumor-specific T lymphocytes. Cancer Res..

[165] Chen M., Leng Y., He C., Li X., Zhao L., Qu Y., Wu Y. (2023). Red blood cells: a potential delivery system. J. Nanobiotechnol..

[166] Bernardo M.E., Fibbe W.E. (2013). Mesenchymal stromal cells: sensors and switchers of inflammation. Cell Stem Cell.

[167] Nicolás-Ávila J.Á., Adrover J.M., Hidalgo A. (2017). Neutrophils in homeostasis, immunity, and cancer. Immunity.

[168] Ballesteros I., Hidalgo A. (2025). The neutrophil collective. Cell.

[169] Parackova Z., Zentsova I., Vrabcova P., Klocperk A., Sumnik Z., Pruhova S., Petruzelkova L., Hasler R., Sediva A. (2020). Neutrophil extracellular trap induced dendritic cell activation leads to Th1 polarization in type 1 diabetes. Front. Immunol..

[170] Findlay E.G., Currie A.J., Zhang A., Ovciarikova J., Young L., Stevens H., McHugh B.J., Canel M., Gray M., Milling S.W.F., Campbell J.D.M., Savill J., Serrels A., Davidson D.J. (2019). Exposure to the antimicrobial peptide LL-37 produces dendritic cells optimized for immunotherapy. OncoImmunology.

[171] Yang D., De La Rosa G., Tewary P., Oppenheim J.J. (2009). Alarmins link neutrophils and dendritic cells. Trends Immunol..

[172] Shahzad A., Ni Y., Yang Y., Liu W., Teng Z., Bai H., Liu X., Sun Y., Xia J., Cui K., Duan Q., Xu Z., Zhang J., Yang Z., Zhang Q. (2025). Neutrophil extracellular traps (NETs) in health and disease. Mol. Biomed..

[173] Grisaru-Tal S., Rothenberg MarcE., Munitz A. (2022). Eosinophil–lymphocyte interactions in the tumor microenvironment and cancer immunotherapy. Nat. Immunol..

[174] Chirumbolo S., Bjørklund G., Sboarina A., Vella A. (2018). The role of basophils as innate immune regulatory cells in allergy and immunotherapy. Hum. Vaccines Immunother..

[175] Yang D., Chen Q., Su S.B., Zhang P., Kurosaka K., Caspi R.R., Michalek S.M., Rosenberg H.F., Zhang N., Oppenheim J.J. (2008). Eosinophil-derived neurotoxin acts as an alarmin to activate the TLR2–MyD88 signal pathway in dendritic cells and enhances Th2 immune responses. J. Exp. Med..

[176] Carretero R., Sektioglu I.M., Garbi N., Salgado O.C., Beckhove P., Hämmerling G.J. (2015). Eosinophils orchestrate cancer rejection by normalizing tumor vessels and enhancing infiltration of CD8+ T cells. Nat. Immunol..

[177] Wei J., Mayberry C.L., Lv X., Hu F., Khan T., Logan N.A., Wilson J.J., Sears J.D., Chaussabel D., Chang C.-H. (2024). IL3-Driven T cell–basophil crosstalk enhances antitumor immunity. Cancer Immunol. Res..

[178] Feng B., Bai Z., Zhou X., Zhao Y., Xie Y.-Q., Huang X., Liu Y., Enbar T., Li R., Wang Y., Gao M., Bonati L., Peng M.-W., Li W., Tao B., Charmoy M., Held W., Melenhorst J.J., Fan R., Guo Y., Tang L. (2024). The type 2 cytokine Fc–IL-4 revitalizes exhausted CD8+ T cells against cancer. Nature.

[179] Sobiepanek A., Kuryk Ł., Garofalo M., Kumar S., Baran J., Musolf P., Siebenhaar F., Fluhr J.W., Kobiela T., Plasenzotti R., Kuchler K., Staniszewska M. (2022). The multifaceted roles of mast cells in immune homeostasis, infections and cancers. Indian J. Manag. Sci..

[180] Katsoulis-Dimitriou K., Kotrba J., Voss M., Dudeck J., Dudeck A. (2020). Mast cell functions linking innate sensing to adaptive immunity. Cells.

[181] Numata T., Harada K., Nakae S. (2022). Roles of mast cells in cutaneous diseases. Front. Immunol..

[182] Moyer T.J., Zmolek A.C., Irvine D.J. (2016). Beyond antigens and adjuvants: formulating future vaccines. J. Clin. Investig..

[183] Thomas S.N., Rohner N.A., Edwards E.E. (2016). Implications of lymphatic transport to lymph nodes in immunity and immunotherapy. Annu. Rev. Biomed. Eng..

[184] U.S. Food and Drug Administration (2011). Guidance for industry: potency tests for cellular and gene therapy products. https://www.fda.gov/media/79856/download.

[185] Salmikangas P., Carlsson B., Klumb C., Reimer T., Thirstrup S. (2023). Potency testing of cell and gene therapy products. Front. Med..

[186] Dunn C.M., Kameishi S., Grainger D.W., Okano T. (2021). Strategies to address mesenchymal stem/stromal cell heterogeneity in immunomodulatory profiles to improve cell-based therapies. Acta Biomater..

[187] Gimona M., Brizzi M.F., Choo A.B.H., Dominici M., Davidson S.M., Grillari J., Hermann D.M., Hill A.F., De Kleijn D., Lai R.C., Lai C.P., Lim R., Monguió-Tortajada M., Muraca M., Ochiya T., Ortiz L.A., Toh W.S., Yi Y.W., Witwer K.W., Giebel B., Lim S.K. (2021). Critical considerations for the development of potency tests for therapeutic applications of mesenchymal stromal cell-derived small extracellular vesicles. Cytotherapy.

[188] Depil S., Duchateau P., Grupp S.A., Mufti G., Poirot L. (2020). ‘Off-the-shelf’ allogeneic CAR T cells: development and challenges. Nat. Rev. Drug Discov..

[189] Ren J., Liu X., Fang C., Jiang S., June C.H., Zhao Y. (2017). Multiplex genome editing to generate universal CAR T cells resistant to PD1 inhibition. Clin. Cancer Res..

[190] Galipeau J., Sensébé L. (2018). Mesenchymal stromal cells: clinical challenges and therapeutic opportunities. Cell Stem Cell.

[191] Marrack P., McKee A.S., Munks M.W. (2009). Towards an understanding of the adjuvant action of aluminium. Nat. Rev. Immunol..

[192] Dunn G.P., Old L.J., Schreiber R.D. (2004). The three Es of cancer immunoediting. Annu. Rev. Immunol..

[193] Pardoll D.M. (2012). The blockade of immune checkpoints in cancer immunotherapy. Nat. Rev. Cancer.

[194] Koyama S., Akbay E.A., Li Y.Y., Herter-Sprie G.S., Buczkowski K.A., Richards W.G., Gandhi L., Redig A.J., Rodig S.J., Asahina H., Jones R.E., Kulkarni M.M., Kuraguchi M., Palakurthi S., Fecci P.E., Johnson B.E., Janne P.A., Engelman J.A., Gangadharan S.P., Costa D.B., Freeman G.J., Bueno R., Hodi F.S., Dranoff G., Wong K.-K., Hammerman P.S. (2016). Adaptive resistance to therapeutic PD-1 blockade is associated with upregulation of alternative immune checkpoints. Nat. Commun..

[195] Mougel A., Terme M., Tanchot C. (2019). Therapeutic cancer vaccine and combinations with antiangiogenic therapies and immune checkpoint blockade. Front. Immunol..

[196] Vollmer J., Krieg A.M. (2009). Immunotherapeutic applications of CpG oligodeoxynucleotide TLR9 agonists. Adv. Drug Deliv. Rev..

[197] Bucks C.M., Norton J.A., Boesteanu A.C., Mueller Y.M., Katsikis P.D. (2009). Chronic antigen stimulation alone is sufficient to drive CD8+ T cell exhaustion. J. Immunol..

